# Integrated physiological, proteomic, and phosphoproteomic analyses of 24-epibrassinolide-mediated yield protection under complete submergence during panicle differentiation in rice

**DOI:** 10.3389/fpls.2026.1969548

**Published:** 2026-09-30

**Authors:** Jie Du, Yu Feng, Yizhen Du, Wenxia Wang

**Affiliations:** Department of Biological and Food Engineering, Lyuliang University, Lyuliang, China

**Keywords:** 24-epibrassinolide, complete submergence, panicle differentiation, phosphoproteomics, yield components

## Abstract

Complete submergence during panicle differentiation can severely reduce rice yield, yet the physiological and molecular responses associated with yield protection by 24-epibrassinolide (EBR) remain unclear. A two-year pot experiment evaluated submergence-sensitive Jiyou 3 and submergence-tolerant Nongxiangzhan under a non-submerged control (CK), complete submergence without EBR (SUB), and complete submergence following foliar pretreatment with 0.06, 0.12, or 0.24 mg L^-1^ EBR. Submergence lasted four days. Yield and physiological traits were assessed in both cultivars, whereas tandem mass tag-based proteomic and phosphoproteomic analyses were confined to Nongxiangzhan under CK, SUB, and 0.12 mg L^-1^ EBR (E12). Across cultivars and years, the greatest yield recovery among EBR treatments was observed under E12. Relative to SUB, E12 increased grain yield per plant by 33.79%-42.03%, primarily through higher grains per panicle and seed-setting rate. E12 increased superoxide dismutase and ascorbate peroxidase activities while reducing hydrogen peroxide and malondialdehyde accumulation. This improved redox status coincided with partial recovery of net photosynthetic rate and Rubisco activity. Higher phosphofructokinase, pyruvate decarboxylase, and alcohol dehydrogenase activities indicated greater glycolytic and ethanolic-fermentation capacity. Nitrogen assimilation was partially maintained, and submergence-induced leaf hormone changes were moderated. Proteomic and phosphoproteomic responses converged on photosynthesis, carbon metabolism, redox regulation, and signaling. Integrated analysis identified 14 proteins with concordant abundance and phosphorylation increases, including components of glycolysis, photosynthetic electron transfer, and antioxidant defense, whereas 74.95% of E12-responsive phosphorylation sites occurred without significant abundance changes. EBR-mediated yield preservation was associated primarily with improved redox homeostasis, maintenance of photosynthetic carbon assimilation, and adjustment of carbon metabolism under oxygen limitation, while nitrogen assimilation and hormone regulation may have supported reproductive development. These findings support further multi-environment field evaluation of EBR pretreatment as a candidate management option to reduce yield loss from short-duration complete submergence during rice panicle differentiation.

## 1 Introduction

Rice (*Oryza sativa* L.) provides a staple food for more than half of the global population, making stable production central to global food security (Rezvi et al., 2023). Increasingly frequent extreme weather events and irregular precipitation are intensifying production risks across major crop-growing regions (Aslam et al., 2023). Recent evidence indicates that extreme rainfall has already caused substantial rice yield losses in China (Fu et al., 2023), while field studies in flood-prone Asian production systems demonstrate that transient submergence can markedly destabilize rice yield (Zhou et al., 2020). Flooding encompasses root-zone waterlogging, partial submergence, and complete submergence, which differ in the extent and duration of oxygen limitation imposed on plant tissues (Lin et al., 2024; Haque et al., 2023). Complete submergence is particularly disruptive because simultaneous coverage of the roots and shoots severely restricts gas exchange. The agronomic consequences depend on flood depth, duration, and crop developmental stage (Ohe and Matsuo, 2020). During panicle differentiation, disruption of spikelet initiation and survival can reduce grain number and subsequent seed setting, resulting in yield losses that are difficult to compensate later in the crop cycle (Meng et al., 2022). Protecting rice from short episodes of complete submergence during this stage is therefore an important target for stress-management research.

Complete submergence disrupts several interdependent physiological processes rather than imposing a single metabolic limitation. Slow diffusion of O_2_ and CO_2_ underwater restricts internal aeration and photosynthetic carbon supply (Leon et al., 2021; Pucciariello, 2020). The resulting oxygen limitation suppresses mitochondrial oxidative phosphorylation and increases reliance on glycolysis coupled with fermentation for ATP production (Alam et al., 2020; Boamfa et al., 2003). Ethanolic fermentation regenerates NAD^+^ and thereby supports continued glycolysis. However, prolonged oxygen deficiency accelerates carbohydrate depletion, while an imbalance between lactate and ethanol fermentation may contribute to cytosolic acidification and metabolic injury (Nurrahma et al., 2022; Basu et al., 2021). Underwater CO_2_ limitation, chlorophyll loss, and impaired photosynthetic metabolism further restrict carbon assimilation and subsequent recovery (Siddiqui et al., 2021; Alpuerto et al., 2022). Redox pressure develops throughout the submergence and de-submergence sequence. Reoxygenation and reillumination after de-submergence can impose additional oxidative stress, while insufficient antioxidant capacity promotes lipid peroxidation, membrane leakage, and delayed recovery (Wang and Komatsu, 2022; Mittal et al., 2022). These metabolic constraints interact with nutrient availability, including nitrogen supply and assimilation, which influence rice recovery following submergence (Xu et al., 2020). Submergence also alters hormone-related processes governing growth restraint, senescence, and leaf recovery (Nolan et al., 2020). During panicle differentiation, the combined disruption of carbon supply, nitrogen metabolism, redox balance, and developmental signaling may compromise spikelet formation and subsequent seed setting. Yield loss at this stage therefore reflects interacting metabolic and developmental constraints rather than failure of a single physiological process.

Brassinosteroids (BRs) are steroid hormones that integrate plant growth and development with environmental stress responses through interactions with redox and phytohormone signaling networks (Wang et al., 2020; Shahzad et al., 2021). Exogenous application of the bioactive BR 24-epibrassinolide (EBR) has been associated with the maintenance of CO_2_ assimilation and photosystem performance, enhancement of enzymatic and non-enzymatic antioxidant defenses, and alteration of stress-responsive hormone profiles across crop species (Ma and Guo, 2014; Pereira et al., 2020). Under waterlogging or root-zone hypoxia, EBR has also been reported to preserve photosynthetic function, limit ROS accumulation and lipid peroxidation, and regulate carbohydrate status and fermentation-related enzyme activities (Kang et al., 2009; Schmitz et al., 2013; Sun et al., 2024). In rice, SUB1A-mediated submergence tolerance involves differential regulation of BR metabolism and signaling, together with BR-gibberellin interactions that restrict shoot elongation during submergence (Zhou et al., 2023). Nevertheless, most studies of exogenous EBR under oxygen-limited conditions have used seedlings or vegetative tissues and have focused primarily on physiological responses. Whether EBR pretreatment can protect yield formation during panicle differentiation under complete submergence has not been established. Proteomic and phosphoproteomic analyses offer complementary means of examining the associated molecular responses. Proteomics characterizes changes in protein abundance across biological processes, whereas phosphoproteomics detects site-specific phosphorylation changes that may occur independently of protein abundance. This distinction is particularly relevant because phosphorylation is integral to BR signaling and plant low-oxygen responses (Cho et al., 2016; Hou et al., 2017). Integrating the two datasets can distinguish abundance-dependent responses from phosphorylation changes and identify proteins affected at both levels. It remains unclear how changes in protein abundance and phosphorylation relate to the physiological and yield responses to EBR pretreatment under complete submergence during panicle differentiation.

The present study tested the hypothesis that foliar EBR pretreatment mitigates yield loss caused by complete submergence during panicle differentiation through coordinated protection of redox and metabolic functions. Two rice cultivars contrasting in submergence tolerance were treated with three EBR concentrations before complete submergence. Grain yield and its components were evaluated alongside photosynthetic performance, oxidative damage and antioxidant defenses, low-oxygen carbon metabolism, nitrogen assimilation, and leaf hormone profiles. TMT-based proteomic and phosphoproteomic analyses were restricted to leaves of the submergence-tolerant cultivar NXZ under the CK, SUB, and 0.12 mg L^-1^ EBR (E12) treatments. Integration of the two omics datasets was used to identify proteins exhibiting changes in abundance, site-specific phosphorylation, or both, and to determine the biological processes represented by these responses. By integrating yield and physiological responses in both cultivars with molecular responses in NXZ, this study aimed to identify candidate processes associated with EBR-mediated protection against complete submergence.

## 2 Materials and methods

### 2.1 Plant materials and cultivation management

Two rice cultivars contrasting in submergence tolerance were used: Jiyou 3 (JY3), a submergence-sensitive cultivar, and Nongxiangzhan (NXZ), a submergence-tolerant cultivar. Both cultivars had a growth period of approximately 118 days. Pot experiments were conducted during the 2023 and 2024 growing seasons in Ronghe Town, Yuncheng, Shanxi Province, China (35°35′55.11″ N, 110°55′24.26″ E). Plastic pots measuring 28 cm in height, 36 cm in top diameter, and 25 cm in bottom diameter were used. Topsoil collected from the 0 to 20 cm layer of a local paddy field was air-dried, crushed using an HY-350 crusher (Haoyun Machinery, Zhengzhou, China), and passed through a 100-mesh sieve. Each pot was filled with 12 kg of the processed soil and maintained under flooded conditions for two weeks before transplanting. Seeds were sown on April 15 in both years, and uniform seedlings were transplanted on May 30 at a density of three hills per pot, with one seedling per hill. Before transplanting, 5 g of compound fertilizer (N:P:K = 15:15:15) was incorporated into the upper 5 cm of soil in each pot as a basal fertilizer. At 7 days after transplanting, 2 g of urea (N ≥ 46.0%, w/w) and 2 g of potassium chloride (K_2_O ≥ 62.0%, w/w) were applied to each pot as tillering fertilizers. All pots received identical nutrient, water, and pest management before treatment application, following standard local practices for high-yield rice cultivation. The physicochemical properties of the experimental soil are provided in **Supplementary Table S1**.

### 2.2 Experimental design

The experiment was conducted during the 2023 and 2024 growing seasons using a two-factor randomized complete block design, with cultivar and treatment as the experimental factors. Five treatments were established for each cultivar. CK comprised non-submerged plants sprayed with the vehicle solution, whereas SUB comprised completely submerged plants sprayed with the same vehicle. E06, E12, and E24 comprised completely submerged plants sprayed with 0.06, 0.12, and 0.24 mg L^-1^ 24-epibrassinolide (EBR; Aladdin, Shanghai, China), respectively. Each cultivar × treatment combination comprised three biological replicates, each consisting of 15 pots. On the two consecutive evenings preceding submergence (18:00-19:00), plants under CK and SUB were uniformly sprayed with the vehicle solution, consisting of water containing 0.1% (v/v) Tween-20. Plants under E06, E12, and E24 were sprayed with the corresponding EBR solutions. All spray solutions contained 0.1% (v/v) Tween-20 and were applied at 10 mL per plant. Complete submergence was initiated on July 5 at the panicle differentiation stage and maintained for four consecutive days. Pots under SUB, E06, E12, and E24 were placed in water tanks measuring 1.5 m × 1.2 m × 1.2 m. The water depth was maintained at 90 cm, ensuring that the uppermost leaf tips and entire shoots remained below the water surface throughout the treatment period. CK plants were maintained under non-submerged conditions during the same period. After four days of complete submergence, the pots were removed from the tanks at approximately 08:00. The leaves were gently rinsed to remove surface sediment, and the plants were transferred to a shaded area for subsequent leaf sampling. Leaf samples for biochemical assays were collected from both cultivars during the first hour after de-submergence, with sampling completed by approximately 09:00. Following sampling, all treatments were maintained under identical conditions. Irrigation was withheld from all pots five days before final harvest.

### 2.3 Determination of yield and yield components

At full maturity in both 2023 and 2024, 10 representative plants were randomly sampled from each replicate for yield analysis. The harvested plants were naturally air-dried to a constant weight. Agronomic traits evaluated included grain yield per plant, effective panicles per plant, total grains per panicle, seed-setting rate, panicle length, and 1000-grain weight.

### 2.4 Determination of photosynthetic characteristics

In 2024, gas-exchange parameters and SPAD values were measured across all treatments between 09:00 and 11:00 on the day of de-submergence, following completion of leaf sampling. Net photosynthetic rate (Pn) and stomatal conductance (Gs) were measured on the middle section of the second fully expanded leaf using a CI-340 portable photosynthesis system (CID Bio-Science, Camas, WA, USA). Five tagged plants were evaluated per replicate. On these same leaves, the relative chlorophyll content (SPAD value) was recorded using a SPAD-502 meter (Konica Minolta, Tokyo, Japan). For Rubisco activity, 0.5 g of frozen leaf tissue collected and stored as described in Section 2.5 was extracted, and the activity was determined by monitoring NADH oxidation at 340 nm utilizing a UV-2600 spectrophotometer (Shimadzu, Kyoto, Japan).

### 2.5 Determination of oxidative stress markers

In 2024, the second fully expanded leaves used for biochemical extraction were collected during the first hour after de-submergence, as described in Section 2.2. Samples were immediately frozen in liquid nitrogen and subsequently stored at -80 °C until analysis. For enzyme and metabolite extraction, 0.25 g of frozen leaf tissue was homogenized in 6 mL of pre-chilled phosphate buffer (0.05 mol L^-1^, pH 8.0, containing 1% PVP) using 3 mm zirconia beads in a high-throughput tissue grinder (60 Hz for 30 s). The homogenate was centrifuged at 4000 × g for 20 min at 4 °C (Eppendorf 5810R, Hamburg, Germany), and the resulting supernatant was collected for subsequent analyses. The contents of malondialdehyde (MDA) and hydrogen peroxide (H_2_O_2_) were quantified using specific ELISA kits (Suzhou Yanxi Biotechnology, Suzhou, China), with absorbance recorded on a Bio-Rad Model 680 microplate reader (Hercules, CA, USA). Superoxide anion (O_2_·^-^) content was determined using a hydroxylamine-based colorimetric assay kit (Suzhou Yanxi Biomedical Technology Co., Ltd., Suzhou, China). Nitrite generated by the reaction of O_2_·^-^ with hydroxylamine hydrochloride was reacted with sulfanilic acid and α-naphthylamine to produce a red azo compound, and absorbance was measured at 530 nm (Liang et al., 2025). Blank-corrected absorbance was converted to O_2_·^-^ content using the manufacturer-provided calibration equation, accounting for the 2:1 stoichiometric ratio of superoxide to nitrite, extraction volume, and sample dilution. Results were normalized to tissue fresh weight (FW) and expressed as nmol g^-1^ FW. For relative electrical conductivity (REC), fresh leaves were collected separately from plants under the corresponding treatments and used without freezing. For each sample, 0.5 g of leaf discs were immersed in 20 mL of deionized water and incubated at 25 °C for 2 h to measure the initial conductivity (C1) using a DDS-307 meter (Shanghai Scientific Instrument, Shanghai, China). The samples were then boiled for 30 min, cooled to room temperature, and measured for final conductivity (C2). REC was calculated as (C1/C2) × 100%.

### 2.6 Analysis of the antioxidant system

Antioxidant parameters were analyzed using the leaf supernatant prepared in Section 2.5. Superoxide dismutase (SOD) and ascorbate peroxidase (APX) activities were determined based on the nitroblue tetrazolium (NBT) photoreduction method (Siqueira et al., 2020) and the protocol described by Kumar (2022), respectively. The contents of reduced ascorbic acid (AsA) and reduced glutathione (GSH) were quantified utilizing the dinitrophenylhydrazine method (Kumar et al., 2011) and the procedure outlined by Giustarini et al. (2013), respectively.

### 2.7 Analysis of carbon and energy metabolism

Using the supernatant obtained in Section 2.5, we quantified the activities of sucrose phosphate synthase (SPS), the glycolytic enzymes phosphofructokinase (PFK) and pyruvate kinase (PK), and the fermentation-related enzymes lactate dehydrogenase (LDH), pyruvate decarboxylase (PDC), and alcohol dehydrogenase (ADH). The concentrations of the intermediate metabolites pyruvate and lactate were also measured. All assays were performed using commercial ELISA kits (Suzhou Yanxi Biotechnology) following the manufacturer’s protocols.

### 2.8 Determination of nitrogen metabolism enzyme activities

The activities of key nitrogen metabolism enzymes, including nitrate reductase (NR), nitrite reductase (NiR), glutamine synthetase (GS), and glutamate synthase (GOGAT), were determined using the leaf supernatant prepared as described in Section 2.5. Assays were conducted using specific ELISA kits (Suzhou Yanxi Biotechnology) with absorbance recorded on a Bio-Rad Model 680 microplate reader.

### 2.9 Determination of endogenous hormones

Leaf samples were collected and stored as described in Section 2.5. Approximately 0.2 g of leaf tissue was extracted with 80% (v/v) methanol at 4 °C. Extracts were purified by petroleum ether washing and ethyl acetate extraction, dried under nitrogen, and reconstituted in 0.1 mL of mobile phase. Abscisic acid (ABA), zeatin (ZA), gibberellin A3 (GA_3_), and indole-3-acetic acid (IAA) were quantified by the Plant Hormone Research Center of China Agricultural University (Beijing, China) using an Agilent 1100 HPLC system with detection at 254 nm. Separation employed a Kromasil C18 column (250 × 4.6 mm, 5 μm) at 30 °C, with 1% aqueous acetic acid/methanol (3:2, v/v) at 0.8 mL min^-1^ and a 10 μL injection volume. Quantification used external calibration curves constructed from the corresponding authentic standards, and concentrations were expressed as ng g^-1^.

### 2.10 Proteomic and phosphoproteomic analysis

In 2024, leaf samples for proteomic and phosphoproteomic analyses were collected from NXZ under CK, SUB, and E12 during the first hour after de-submergence (Section 2.2). For each treatment, samples were obtained from the same three biological replicates used for physiological analyses, with leaves from five individual plants pooled per replicate. Samples for the two analyses were prepared separately and did not share a common TMT-labeled peptide pool. Proteins were extracted using a phenol-based procedure with protease and phosphatase inhibitors, precipitated with ammonium acetate in methanol, washed, and redissolved in 8 M urea. Protein concentrations were determined using a BCA assay. Equal protein amounts were subjected to trypsin digestion at an enzyme-to-protein ratio of 1:50 (w/w), followed by reduction and alkylation. For proteomic analysis, peptides were labeled with TMT reagents (Thermo Fisher Scientific) for 2 h at room temperature. Following assessment of labeling efficiency, equal amounts of labeled peptides were pooled and desalted, with quantification configured for TMT-10plex. High-pH reversed-phase HPLC fractions were combined into 14 fractions for proteomics and nine for phosphoproteomics. Proteomic fractions were analyzed using an EASY-nLC 1000-Q Exactive Plus system, and phosphoproteomic fractions using an EASY-nLC 1200-Orbitrap Exploris 480 system (Thermo Fisher Scientific). Both analyses employed 60 min gradients at 500 nL min^-1^ in data-dependent acquisition mode. MS/MS resolutions were 35,000 and 15,000, respectively.

Raw data were processed using MaxQuant software (v1.6.15.0) against *Oryza sativa* subsp. *indica* databases containing 37,383 sequences for proteomics (20250815 version) and 63,173 sequences for phosphoproteomics (20200713 version), supplemented with reverse and contaminant sequences. Searches used Trypsin/P specificity, allowing two missed cleavages, with fixed cysteine carbamidomethylation. The FDR was controlled at 1% at the protein and peptide-spectrum match levels. Relative quantification used TMT reporter-ion intensities. For proteomics, peptide intensities were divided by their respective means across samples and median-normalized within each sample. Protein abundance was calculated as the median normalized abundance of the corresponding unique peptides. For phosphoproteomics, phosphorylation-site intensities were divided by their respective means across samples. Differentially expressed proteins (DEPs) and differentially phosphorylated sites (DPSs) were identified based on a fold change (FC) > 1.3 or < 0.77, with statistical significance set at *P* < 0.05 (Student’s t-test). Functional enrichment analyses, including Gene Ontology (GO) and Kyoto Encyclopedia of Genes and Genomes (KEGG) pathways, were performed using the clusterProfiler package in R, utilizing annotations from RAP-DB.

### 2.11 RNA extraction, RNA sequencing, and qRT-PCR analysis

Total RNA was extracted from the same leaf tissues used for proteomic analysis using the RNAprep Pure Plant Kit (Tiangen Biotech, Beijing, China), and its quality was assessed by agarose gel electrophoresis and a NanoDrop 2000 spectrophotometer (Thermo Fisher Scientific). RNA-seq was performed on nine NXZ leaf samples under CK, SUB, and E12, with three biological replicates each. Libraries were prepared by RNA fragmentation, cDNA synthesis, adaptor ligation, PCR amplification, and circularization, followed by DNBSEQ sequencing. Reads were filtered using Trimmomatic v0.36 and aligned to the *Oryza sativa* subsp. *indica* reference genome from Ensembl release 48 using HISAT2 v2.1.0. For expression quantification, clean reads were aligned to reference gene sequences using Bowtie2, and gene expression levels were estimated using RSEM. Six genes encoding proteins with concordant increases in abundance and phosphorylation were selected for qRT-PCR analysis. Their sequences were used for primer design, and their RNA-seq expression changes under E12 relative to SUB were compared with the qRT-PCR results. Raw sequencing data are available in the NCBI Sequence Read Archive under BioProject accession PRJNA1418166. For qRT-PCR, 1 μg of total RNA was reverse-transcribed using the PrimeScript™ RT reagent Kit with gDNA Eraser (Takara Bio, Shiga, Japan). Reactions were performed in a 20 μL volume using TB Green^®^ Premix Ex Taq™ II (Takara Bio) on a CFX96 system (Bio-Rad Laboratories, Hercules, CA, USA). Relative expression was calculated using the 2^-ΔΔ^*^Ct^* method with *OsActin1* (GenBank: XM_015773579.2) as the reference gene and SUB as the calibrator. Three biological replicates were analyzed per treatment, with three technical replicates per biological sample. Primer sequences are provided in **Supplementary Table S2**.

### 2.12 Statistical analysis

Analyses were performed using IBM SPSS Statistics 22.0 (IBM Corp., Armonk, NY, USA). Data are means ± SD (n = 3). Physiological and biochemical traits were analyzed by two-way ANOVA for cultivar, treatment, and their interaction. Duncan’s test compared treatments within cultivars and cultivars within treatments using the ANOVA residual mean square (*P* < 0.05).

## 3 Results

### 3.1 Grain yield and yield components

Across the 2023 and 2024 growing seasons, complete submergence markedly reduced grain yield in both rice cultivars (**Table 1**). Relative to CK, SUB decreased per-plant grain yield by 54.07% and 57.31% in JY3 and by 46.09% and 50.25% in NXZ in 2023 and 2024, respectively. These yield losses were accompanied by significant reductions in effective panicles per plant, grains per panicle, and seed-setting rate in both cultivars and years. Panicle length was significantly reduced only in JY3 in 2024, whereas thousand-grain weight did not differ significantly among treatments in either cultivar or year. EBR pretreatment partially alleviated the yield loss, with E12 producing the highest per-plant grain yield among the submerged treatments in both cultivars and years. Compared with SUB, E12 increased per-plant grain yield by 36.01% and 42.03% in JY3 and by 33.79% and 38.02% in NXZ in 2023 and 2024, respectively. The yield response was accompanied by increases in grains per panicle of 15.18% and 16.84% in JY3 and 14.34% and 16.42% in NXZ, together with increases in seed-setting rate of 11.01% and 12.46% in JY3 and 9.45% and 9.85% in NXZ. In both years, E12 significantly increased per-plant grain yield, grains per panicle, and seed-setting rate relative to SUB in each cultivar, but did not significantly affect effective panicle number, panicle length, or thousand-grain weight. The increases in per-plant grain yield, grains per panicle, and seed-setting rate under E06 and E24 were smaller than those under E12.

**Table 1:** Effects of EBR pretreatment on grain yield and yield components of two rice cultivars under complete submergence.

Years	Variety	Treatment	Panicle length (cm)	Effective panicle	Total grains per panicle	Seed setting rate (%)	1000-grain weight(g)	Yieldper plant (g)
2023	JY3	CK	20.06 ± 0.64a	7.60 ± 0.32a	133.35 ± 4.62a	83.32 ± 2.22a	23.70 ± 0.44a	20.01 ± 0.86a
		SUB	18.98 ± 0.57a	6.38 ± 0.31b	97.15 ± 3.08c	64.95 ± 1.87c	23.18 ± 0.35a	9.33 ± 0.64d
		E6	19.05 ± 0.58a	6.50 ± 0.27b	103.50 ± 3.87bc	68.00 ± 1.95bc	23.22 ± 0.41a	10.62 ± 0.76cd
		E12	19.33 ± 0.60a	6.74 ± 0.34b	111.90 ± 3.51b	72.10 ± 2.04b	23.33 ± 0.37a	12.69 ± 0.71b
		E24	19.16 ± 0.59a	6.58 ± 0.30b	106.80 ± 4.02bc	69.80 ± 1.99bc	23.27 ± 0.46a	11.41 ± 0.80bc
	NXZ	CK	18.26 ± 0.47a	8.15 ± 0.40a	140.80 ± 4.37a	81.50 ± 2.47a	24.59 ± 0.47a	23.00 ± 0.94a
		SUB	17.63 ± 0.55a	7.22 ± 0.33b	102.50 ± 3.16c	68.80 ± 1.84c	24.36 ± 0.40a	12.40 ± 0.72c
		E6	17.72 ± 0.49a	7.37 ± 0.35ab	108.00 ± 3.82bc	71.20 ± 2.18bc	24.45 ± 0.44a	13.86 ± 0.84c
		E12	17.83 ± 0.57a	7.65 ± 0.37ab	117.20 ± 3.51b	75.30 ± 2.03b	24.58 ± 0.49a	16.59 ± 0.76b
		E24	17.75 ± 0.51a	7.40 ± 0.32ab	109.00 ± 3.68bc	71.40 ± 2.25bc	24.50 ± 0.42a	14.11 ± 0.81c
2024	JY3	CK	20.42 ± 0.69a	7.78 ± 0.37a	135.20 ± 4.18a	83.00 ± 2.36a	23.74 ± 0.41a	20.73 ± 0.91a
		SUB	18.63 ± 0.63b	6.25 ± 0.26b	95.00 ± 3.95c	64.20 ± 2.08c	23.22 ± 0.46a	8.85 ± 0.68c
		E6	19.10 ± 0.55ab	6.47 ± 0.31b	102.00 ± 3.34bc	67.50 ± 2.17bc	23.24 ± 0.38a	10.35 ± 0.74c
		E12	19.45 ± 0.66ab	6.72 ± 0.35b	111.00 ± 4.05b	72.20 ± 2.02b	23.34 ± 0.42a	12.57 ± 0.78b
		E24	19.14 ± 0.61ab	6.48 ± 0.28b	104.00 ± 3.76bc	69.30 ± 2.26bc	23.28 ± 0.35a	10.87 ± 0.77c
	NXZ	CK	18.48 ± 0.52a	8.28 ± 0.36a	142.50 ± 4.11a	81.80 ± 2.29a	24.60 ± 0.43a	23.74 ± 0.89a
		SUB	17.35 ± 0.48a	7.10 ± 0.30b	100.50 ± 3.46c	68.00 ± 2.05c	24.35 ± 0.37a	11.81 ± 0.76c
		E6	17.80 ± 0.55a	7.30 ± 0.38b	107.50 ± 3.91bc	71.40 ± 1.88bc	24.40 ± 0.46a	13.67 ± 0.82c
		E12	17.90 ± 0.52a	7.60 ± 0.34ab	117.00 ± 4.10b	74.70 ± 2.15b	24.54 ± 0.41a	16.30 ± 0.87b
		E24	17.60 ± 0.57a	7.32 ± 0.32b	108.50 ± 3.58bc	70.80 ± 2.12bc	24.38 ± 0.39a	13.71 ± 0.73c

CK, non-submerged control; SUB, complete submergence without EBR; E06, E12, and E24, complete submergence following foliar pretreatment with 0.06, 0.12, and 0.24 mg L^-1^ EBR, respectively. Values are means ± SD (n = 3). Within each year and cultivar, different lowercase letters within a column indicate significant differences among treatments (*P* < 0.05).

### 3.2 Photosynthetic traits, oxidative damage, and antioxidant responses

Complete submergence significantly impaired photosynthetic performance in both rice cultivars (**Figure 1**). Two-way ANOVA showed significant effects of cultivar and treatment on all four parameters (*P* < 0.05), whereas their interaction was not significant. Relative to CK, SUB reduced the net photosynthetic rate (Pn) by 39.32% in JY3 and 32.08% in NXZ (**Figure 1A**). Stomatal conductance (Gs) decreased by 27.28% and 28.44%, respectively (**Figure 1B**). Rubisco activity fell by 39.41% in JY3 and 30.05% in NXZ (**Figure 1C**), while SPAD values declined by 23.39% and 22.39% (**Figure 1D**). E12 produced the highest values for all four parameters among the submerged treatments in both cultivars. Compared with SUB, E12 increased Pn by 34.40% in JY3 and 22.22% in NXZ, together with increases of 16.06% and 17.02% in Gs. Rubisco activity increased by 24.80% and 18.77%, whereas SPAD values increased by 5.42% and 13.33% (**Figures 1A–D**). In JY3, E12 significantly increased Pn, Gs, and Rubisco activity relative to SUB, but did not significantly affect SPAD. In NXZ, all four parameters were significantly higher under E12 than under SUB. E12 and E24 did not differ significantly in any photosynthetic parameter except Pn in JY3, which was higher under E12. Within-treatment comparisons showed that NXZ had significantly higher Pn and Rubisco activity than JY3 under SUB, higher Gs under CK, and higher SPAD values under E06, E12, and E24 (**Figures 1A–D**).

**Figure 1:**
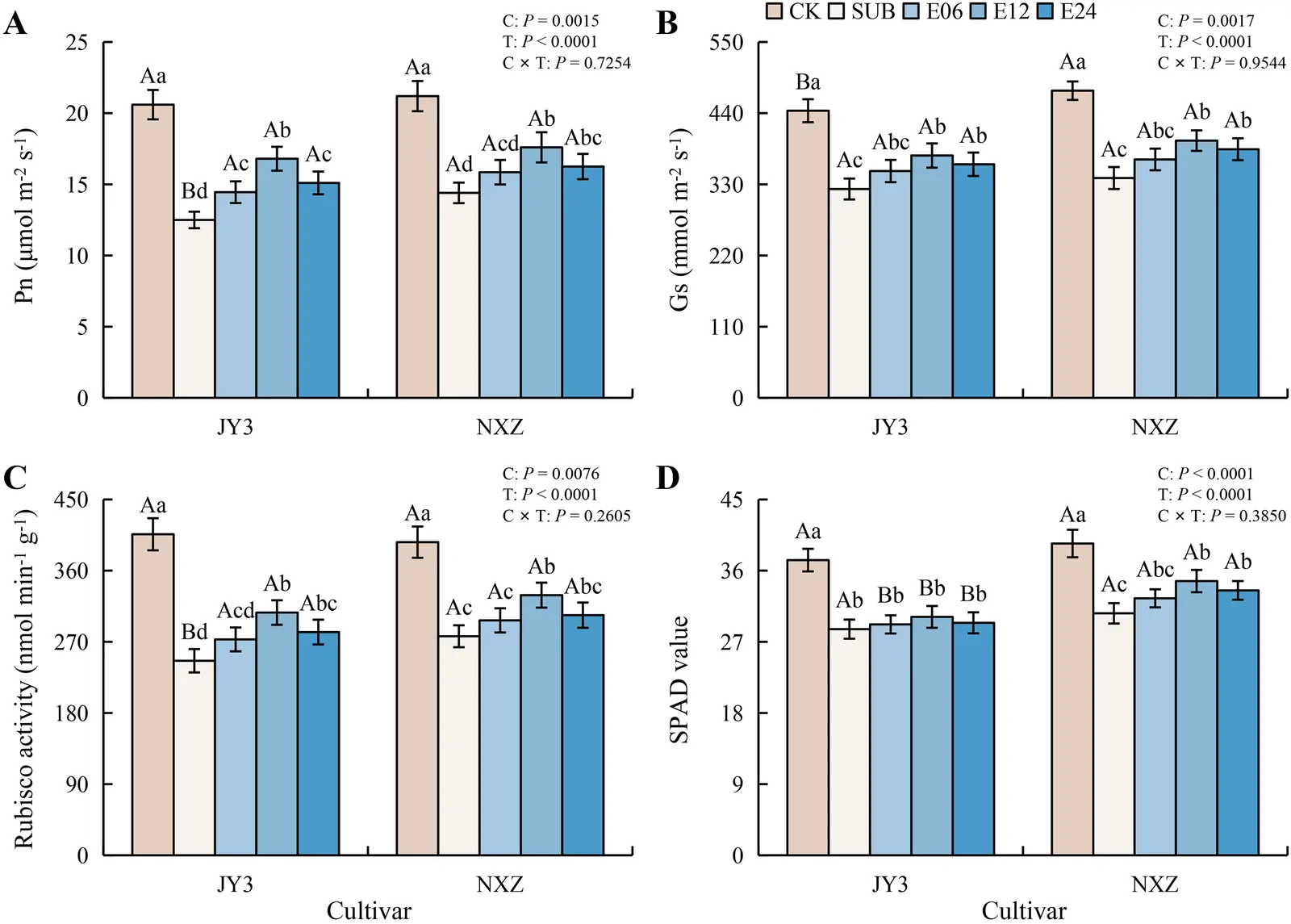
Effects of EBR pretreatment on photosynthetic performance in rice under complete submergence. **(A)** Net photosynthetic rate (Pn), **(B)** stomatal conductance (Gs), **(C)** Rubisco activity, and **(D)** SPAD value. JY3, Jiyou 3; NXZ, Nongxiangzhan. CK, non-submerged control; SUB, complete-submergence control; E06, E12, and E24, complete submergence following pretreatment with 0.06, 0.12, and 0.24 mg L^-1^ EBR, respectively. Data are means ± SD (n = 3). Lowercase and uppercase letters denote comparisons among treatments within each cultivar and between cultivars within each treatment, respectively. Means sharing a letter within the corresponding comparison do not differ significantly (Duncan’s test, *P* < 0.05). C, T, and C × T denote cultivar, treatment, and their interaction in two-way ANOVA.

Complete submergence caused pronounced ROS accumulation and membrane injury (**Figure 2**). Two-way ANOVA showed significant effects of cultivar and treatment on all four indicators (*P* < 0.05). The cultivar × treatment interaction was significant for H_2_O_2_ content (*P* < 0.05) and relative electrical conductivity (*P* < 0.05), but not for O_2_·^-^ or MDA contents. Compared with CK, SUB increased O_2_·^-^ levels by 70.54% in JY3 and 67.41% in NXZ (**Figure 2A**). H_2_O_2_ contents rose by 74.83% and 40.17% (**Figure 2B**), while MDA contents increased by 68.92% and 54.65% (**Figure 2C**). Relative electrical conductivity exhibited the largest proportional changes, increasing by 330.51% in JY3 and 296.19% in NXZ (**Figure 2D**). EBR pretreatment alleviated these effects, with E12 producing the lowest values for all four indicators among the submerged treatments. Relative to SUB, E12 lowered ROS accumulation in both cultivars: O_2_·^-^ levels declined by 27.03% in JY3 and 27.72% in NXZ, while H_2_O_2_ contents decreased by 21.82% and 14.75% (**Figures 2A, B**). Membrane injury was likewise alleviated, as MDA content decreased by 27.21% in JY3 and 23.35% in NXZ, and relative electrical conductivity fell by 36.61% and 37.26% (**Figures 2C, D**). All four indicators were significantly lower under E12 than under SUB. Within-treatment comparisons showed that JY3 had significantly higher O_2_·^-^, H_2_O_2_, and MDA contents and relative electrical conductivity than NXZ under SUB and all three EBR treatments, whereas cultivar differences were not significant under CK (**Figures 2A–D**).

**Figure 2:**
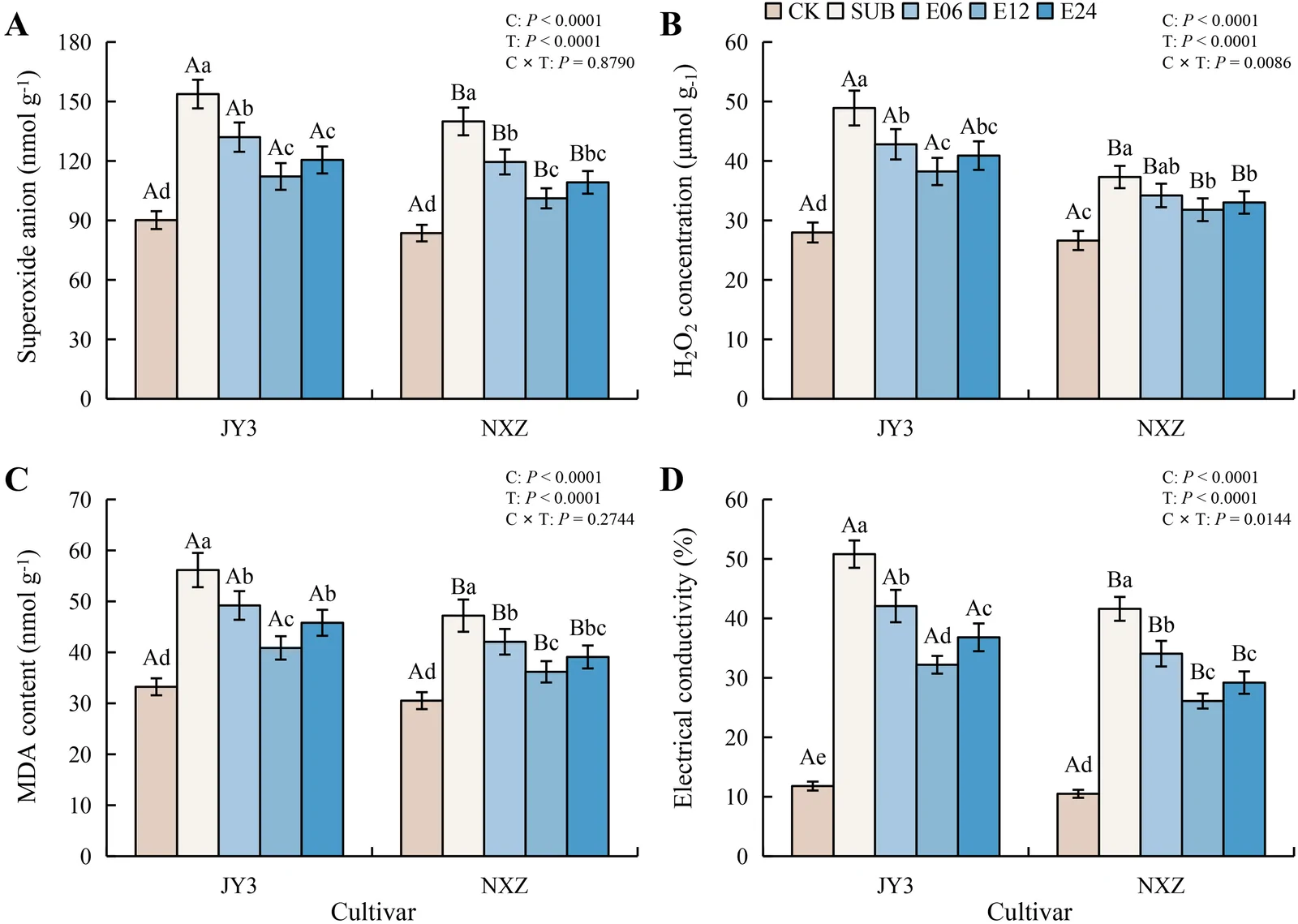
Effects of EBR pretreatment on reactive oxygen species accumulation and membrane damage in rice under complete submergence. **(A)** Superoxide anion (O_2_·^-^) content, **(B)** hydrogen peroxide (H_2_O_2_) content, **(C)** malondialdehyde (MDA) content, and **(D)** relative electrical conductivity. CK, non-submerged control; SUB, complete-submergence control; E06, E12, and E24, complete submergence following pretreatment with 0.06, 0.12, and 0.24 mg L^-1^ EBR, respectively. Data are means ± SD (n = 3). Lowercase and uppercase letters denote comparisons among treatments within each cultivar and between cultivars within each treatment, respectively. Means sharing a letter within the corresponding comparison do not differ significantly (Duncan’s test, *P* < 0.05). C, T, and C × T denote cultivar, treatment, and their interaction in two-way ANOVA.

The accumulation of ROS and membrane damage under complete submergence was accompanied by significant increases in enzymatic and non-enzymatic antioxidant components (**Figure 3**). Two-way ANOVA showed significant effects of cultivar and treatment on all four indicators (*P* < 0.05), whereas their interaction was not significant. Under SUB, SOD and APX activities increased by 40.93% and 28.13% in JY3 and by 46.45% and 37.61% in NXZ relative to CK. AsA and GSH contents rose by 31.98% and 31.88% in JY3, with corresponding increases of 33.97% and 32.10% in NXZ. EBR pretreatment further enhanced these antioxidant responses. Relative to SUB, E12 increased SOD and APX activities by 15.73% and 19.97% in JY3 and by 15.63% and 20.85% in NXZ (**Figures 3A, B**). Similar responses were observed for AsA and GSH contents, which increased by 13.69% and 14.29% in JY3 and by 15.19% and 14.02% in NXZ (**Figures 3C, D**). All four antioxidant indicators were significantly higher under E12 than under SUB in both cultivars, with no significant differences among the three EBR concentrations. Within-treatment comparisons showed that NXZ had significantly higher SOD and APX activities and AsA and GSH contents than JY3 under SUB and all three EBR treatments (**Figures 3A–D**). Under CK, only GSH content differed significantly between cultivars, with a higher value in NXZ (**Figure 3D**).

**Figure 3:**
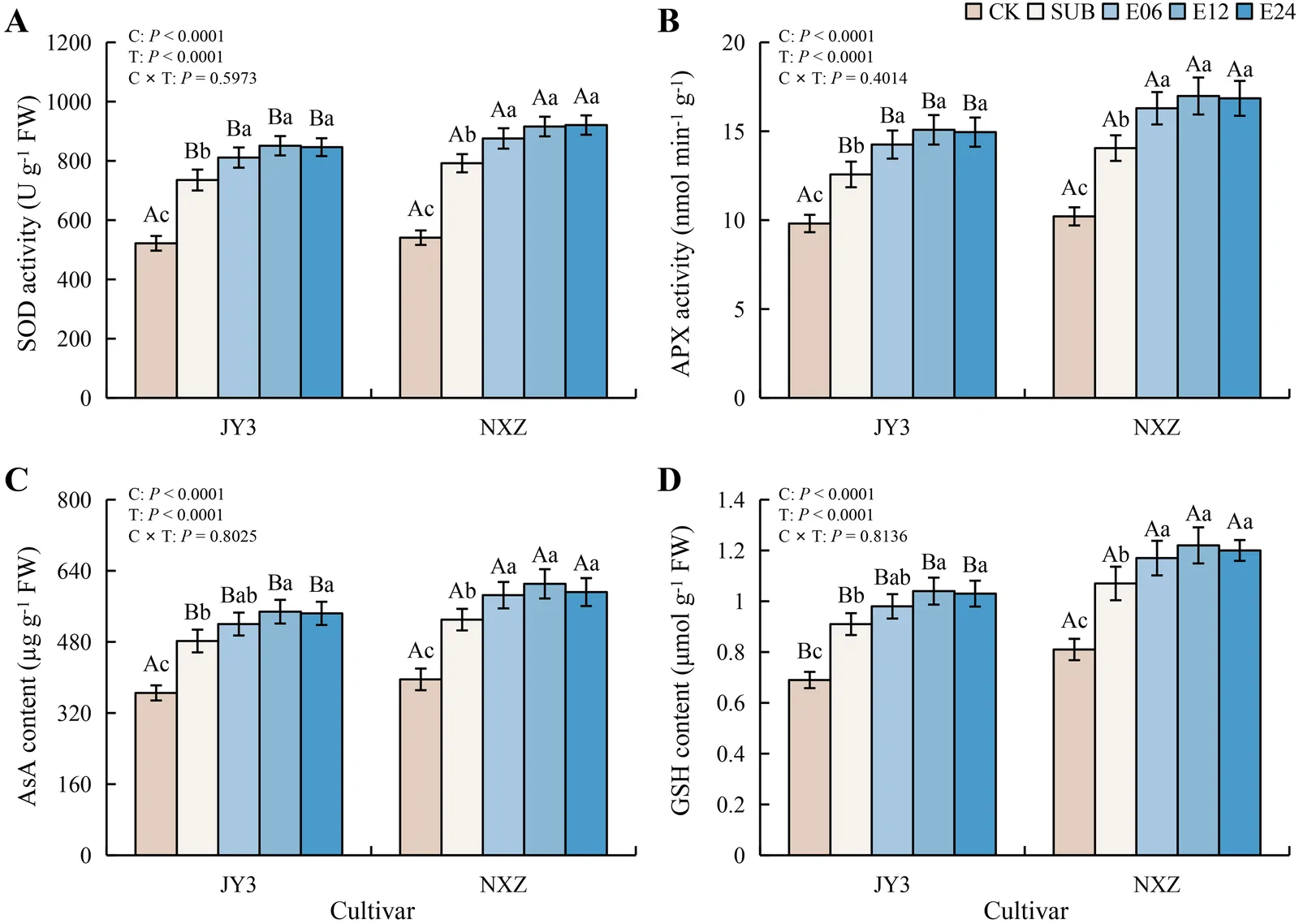
Effects of EBR pretreatment on antioxidant defense in rice under complete submergence. **(A)** Superoxide dismutase (SOD) activity, **(B)** ascorbate peroxidase (APX) activity, **(C)** ascorbic acid (AsA) content, and **(D)** reduced glutathione (GSH) content. Data are means ± SD (n = 3). CK, non-submerged control; SUB, complete-submergence control; E06, E12, and E24, complete submergence following pretreatment with 0.06, 0.12, and 0.24 mg L^-1^ EBR, respectively. Data are means ± SD (n = 3). Lowercase and uppercase letters denote comparisons among treatments within each cultivar and between cultivars within each treatment, respectively. Means sharing a letter within the corresponding comparison do not differ significantly (Duncan’s test, *P* < 0.05). C, T, and C × T denote cultivar, treatment, and their interaction in two-way ANOVA.

### 3.3 Carbon and nitrogen metabolism and leaf hormone contents

Complete submergence markedly altered carbohydrate metabolism and glycolysis in both cultivars (**Figure 4**). Two-way ANOVA showed significant treatment effects on all four indicators (*P* < 0.05). Cultivar effects were significant for SPS, PFK, and PK activities (*P* < 0.05), but not for pyruvate content. The cultivar × treatment interaction was significant only for SPS activity (*P* < 0.05). Relative to CK, SUB reduced sucrose-phosphate synthase (SPS) activity by 39.26% in JY3 and 19.83% in NXZ (**Figure 4A**). Conversely, phosphofructokinase (PFK) activity, pyruvate kinase (PK) activity, and pyruvate content increased by 42.95%, 33.33%, and 30.13% in JY3 (**Figures 4B–D**). NXZ showed corresponding increases of 56.44%, 38.10%, and 27.04%. Among the three EBR concentrations, E12 produced the highest values for all four indicators in both cultivars. Compared with SUB, E12 significantly increased SPS, PFK, and PK activities by 34.18%, 17.89%, and 27.63% in JY3, together with a significant 20.61% increase in pyruvate content. In NXZ, the corresponding significant increases were 22.16%, 20.51%, 29.89%, and 15.99%. Within-treatment comparisons showed that NXZ had significantly higher SPS and PFK activities than JY3 under SUB and all three EBR treatments (**Figures 4A, B**). PK activity was also significantly higher in NXZ than in JY3 under SUB, E12, and E24 (**Figure 4C**).

**Figure 4:**
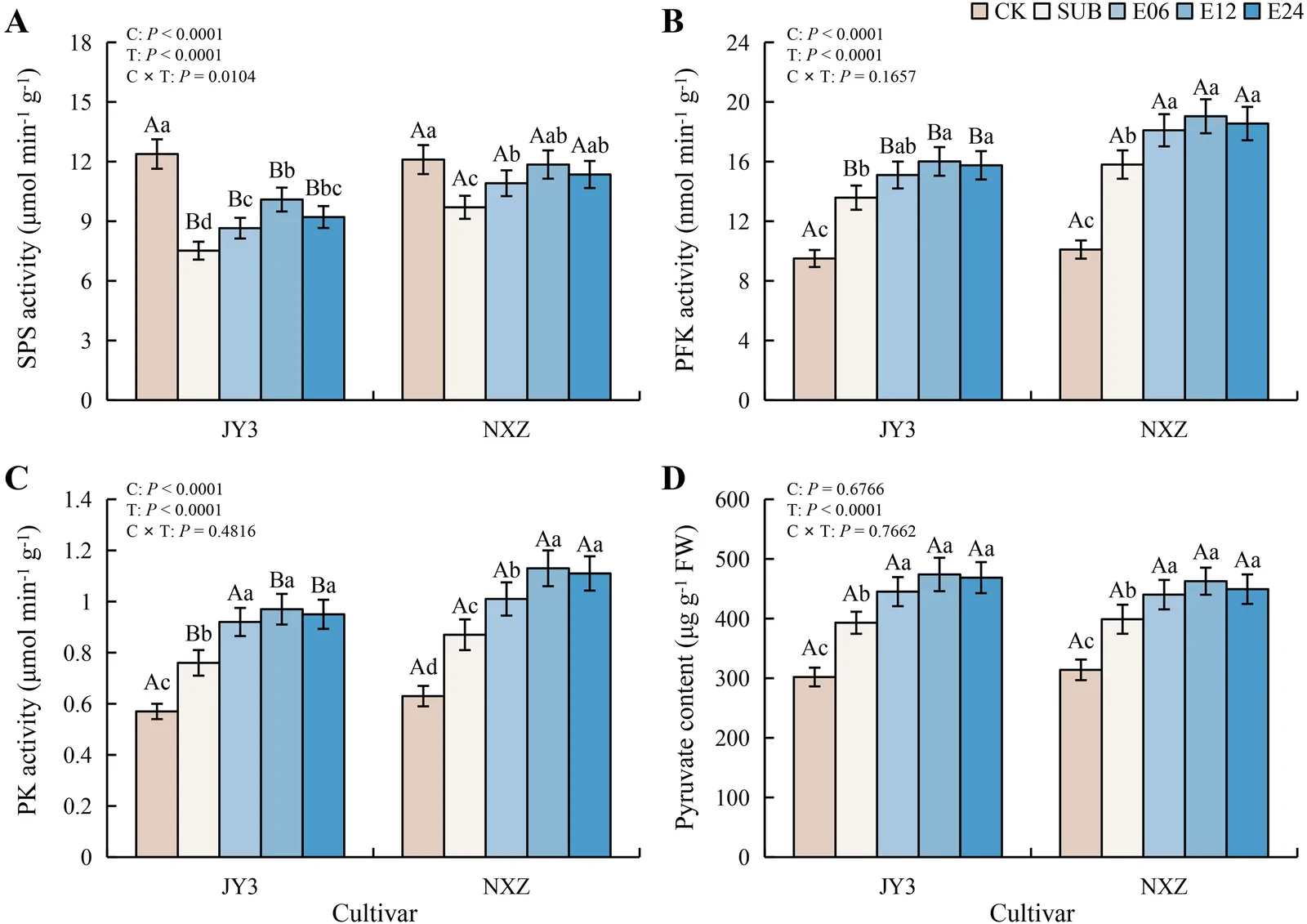
Effects of EBR pretreatment on sucrose metabolism and glycolysis in rice under complete submergence. **(A)** Sucrose-phosphate synthase (SPS) activity, **(B)** phosphofructokinase (PFK) activity, **(C)** pyruvate kinase (PK) activity, and **(D)** pyruvate content. CK, non-submerged control; SUB, complete-submergence control; E06, E12, and E24, complete submergence following pretreatment with 0.06, 0.12, and 0.24 mg L^-1^ EBR, respectively. Data are means ± SD (n = 3). Lowercase and uppercase letters denote comparisons among treatments within each cultivar and between cultivars within each treatment, respectively. Means sharing a letter within the corresponding comparison do not differ significantly (Duncan’s test, *P* < 0.05). C, T, and C × T denote cultivar, treatment, and their interaction in two-way ANOVA.

Complete submergence increased LDH, PDC, and ADH activities and lactate content in both cultivars (**Figure 5**). Two-way ANOVA showed significant treatment effects on all four indicators (*P* < 0.05). Cultivar effects were also significant for LDH activity (*P* < 0.05) and the other three indicators (*P* < 0.05), whereas no significant cultivar × treatment interaction was detected. Under SUB, LDH activity and lactate content increased by 39.32% and 43.94% in JY3 and by 28.72% and 29.69% in NXZ relative to CK (**Figures 5A, B**). PDC and ADH activities increased concurrently, by 72.40% and 47.72% in JY3 and by 74.47% and 58.76% in NXZ (**Figures 5C, D**). Under E12, LDH activity and lactate content decreased, whereas PDC and ADH activities increased relative to SUB. In JY3, the reductions in LDH activity and lactate content were 18.08% and 14.74%, while PDC and ADH activities increased by 23.54% and 17.62%. In NXZ, LDH activity and lactate content decreased by 13.02% and 13.25%, while PDC and ADH activities increased by 36.82% and 18.16%. All four changes were significant in both cultivars. E24 significantly reduced LDH activity and lactate content and increased PDC and ADH activities in both cultivars. Within-treatment comparisons showed that JY3 had significantly higher lactate content than NXZ under SUB and all three EBR treatments (**Figure 5B**). NXZ had significantly higher PDC activity than JY3 under all three EBR treatments and higher ADH activity under SUB and all three EBR treatments (**Figures 5C, D**).

**Figure 5:**
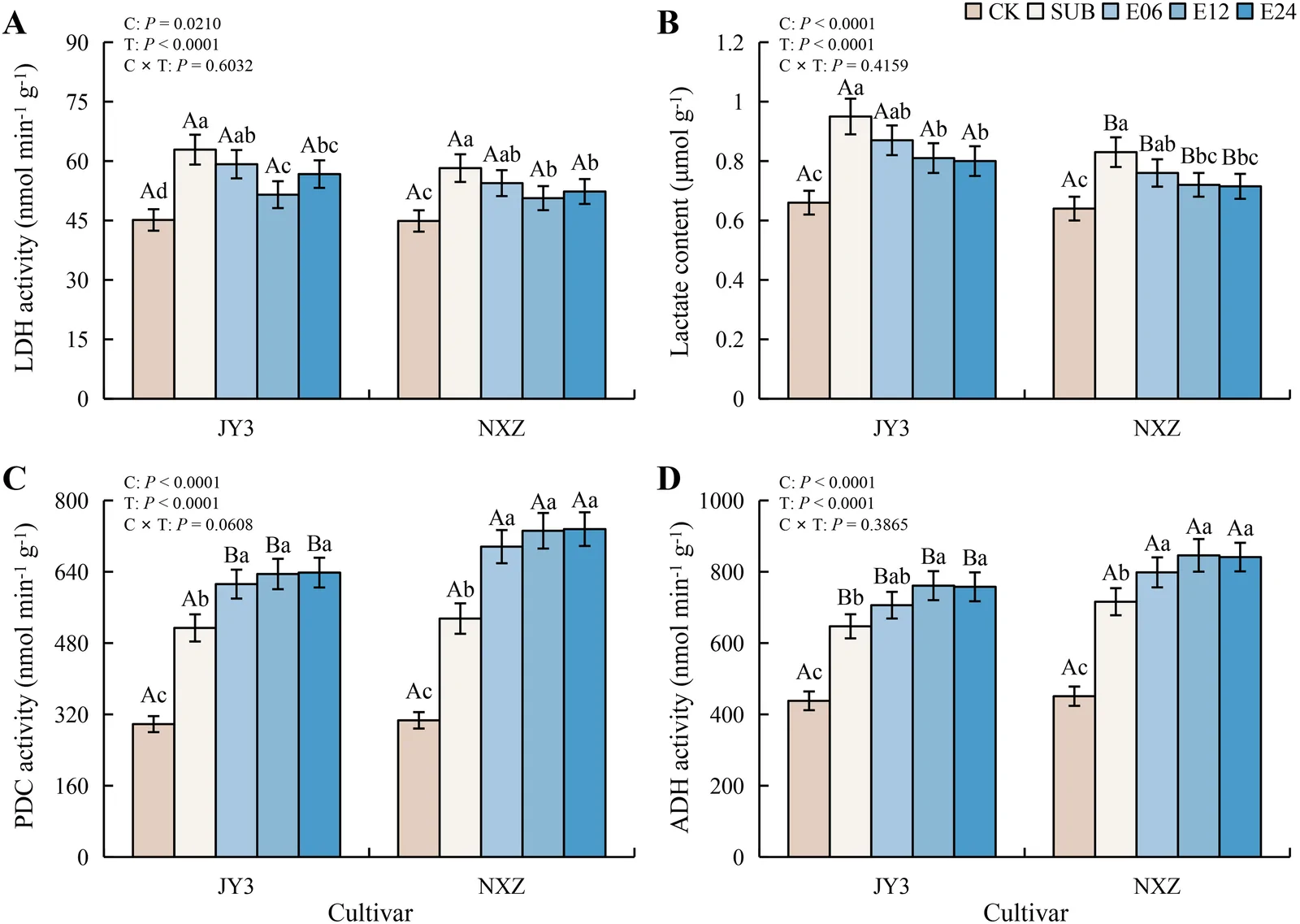
Effects of EBR pretreatment on lactate and ethanolic fermentation in rice under complete submergence. **(A)** Lactate dehydrogenase (LDH) activity, **(B)** lactate content, **(C)** pyruvate decarboxylase (PDC) activity, and **(D)** alcohol dehydrogenase (ADH) activity. CK, non-submerged control; SUB, complete-submergence control; E06, E12, and E24, complete submergence following pretreatment with 0.06, 0.12, and 0.24 mg L^-1^ EBR, respectively. Data are means ± SD (n = 3). Lowercase and uppercase letters denote comparisons among treatments within each cultivar and between cultivars within each treatment, respectively. Means sharing a letter within the corresponding comparison do not differ significantly (Duncan’s test, *P* < 0.05). C, T, and C × T denote cultivar, treatment, and their interaction in two-way ANOVA.

Complete submergence significantly suppressed nitrogen-assimilating enzyme activities in both cultivars (**Figure 6**). Two-way ANOVA showed significant effects of cultivar and treatment on all four enzyme activities (*P* < 0.05). The cultivar × treatment interaction was significant for NR and NiR (*P* < 0.05). Relative to CK, SUB reduced NR, NiR, GS, and GOGAT activities by 51.93%, 47.78%, 44.70%, and 46.67% in JY3 (**Figures 6A–D**). The corresponding reductions in NXZ were 28.87%, 39.78%, 36.17%, and 33.64%. E12 partially counteracted these reductions. In JY3, E12 significantly increased NR, NiR, GS, and GOGAT activities by 47.46%, 38.30%, 34.47%, and 42.44% relative to SUB. The corresponding significant increases in NXZ were 31.64%, 43.64%, 26.67%, and 31.09%. E12 produced significantly higher NR activity than E24 in NXZ, whereas the other three enzyme activities did not differ significantly between these treatments. Within-treatment comparisons showed that NXZ had significantly higher NR, NiR, and GOGAT activities than JY3 under SUB and all three EBR treatments (**Figures 6A, B, D**). GS activity was significantly higher in NXZ than in JY3 under SUB and E06, but did not differ significantly between cultivars under E12 or E24 (**Figure 6C**).

**Figure 6:**
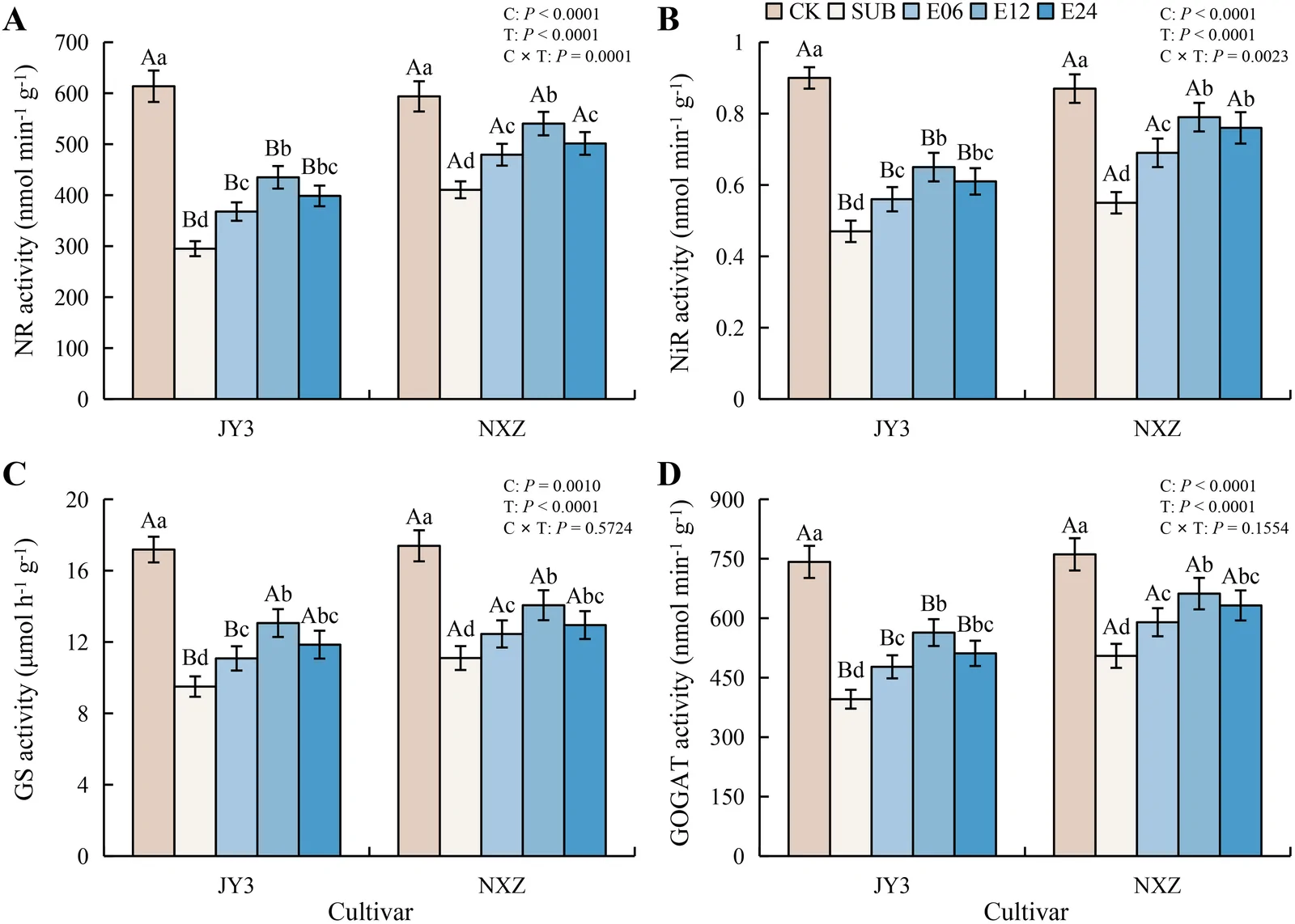
Effects of EBR pretreatment on nitrogen-assimilating enzyme activities in rice under complete submergence. **(A)** Nitrate reductase (NR) activity, **(B)** nitrite reductase (NiR) activity, **(C)** glutamine synthetase (GS) activity, and **(D)** glutamate synthase (GOGAT) activity. CK, non-submerged control; SUB, complete-submergence control; E06, E12, and E24, complete submergence following pretreatment with 0.06, 0.12, and 0.24 mg L^-1^ EBR, respectively. Data are means ± SD (n = 3). Lowercase and uppercase letters denote comparisons among treatments within each cultivar and between cultivars within each treatment, respectively. Means sharing a letter within the corresponding comparison do not differ significantly (Duncan’s test, *P* < 0.05). C, T, and C × T denote cultivar, treatment, and their interaction in two-way ANOVA.

Complete submergence shifted leaf hormone profiles toward higher ABA and IAA contents and lower zeatin and GA_3_ contents (**Figure 7**). Two-way ANOVA showed significant treatment effects on all four hormones (*P* < 0.05). Cultivar effects were significant for zeatin, GA_3_, and IAA contents (*P* < 0.05), but not for ABA content. No significant cultivar × treatment interaction was detected for any hormone. Under SUB, ABA and IAA increased by 36.95% and 32.50% in JY3 relative to CK, while zeatin and GA_3_ decreased by 41.08% and 33.50%. NXZ showed increases of 34.54% in ABA and 20.57% in IAA, accompanied by reductions of 31.76% in zeatin and 27.16% in GA_3_. Compared with SUB, E12 significantly reduced ABA and IAA contents by 14.92% and 17.81% in JY3 and by 15.16% and 13.47% in NXZ (**Figures 7A, D**). E12 also significantly increased zeatin and GA_3_ contents by 29.58% and 17.69% in JY3 and by 23.55% and 16.27% in NXZ (**Figures 7B, C**). Within-treatment comparisons showed that NXZ had significantly higher zeatin, GA_3_, and IAA contents than JY3 under all five treatments (**Figures 7B–D**). ABA content did not differ significantly between cultivars under any treatment (**Figure 7A**).

**Figure 7:**
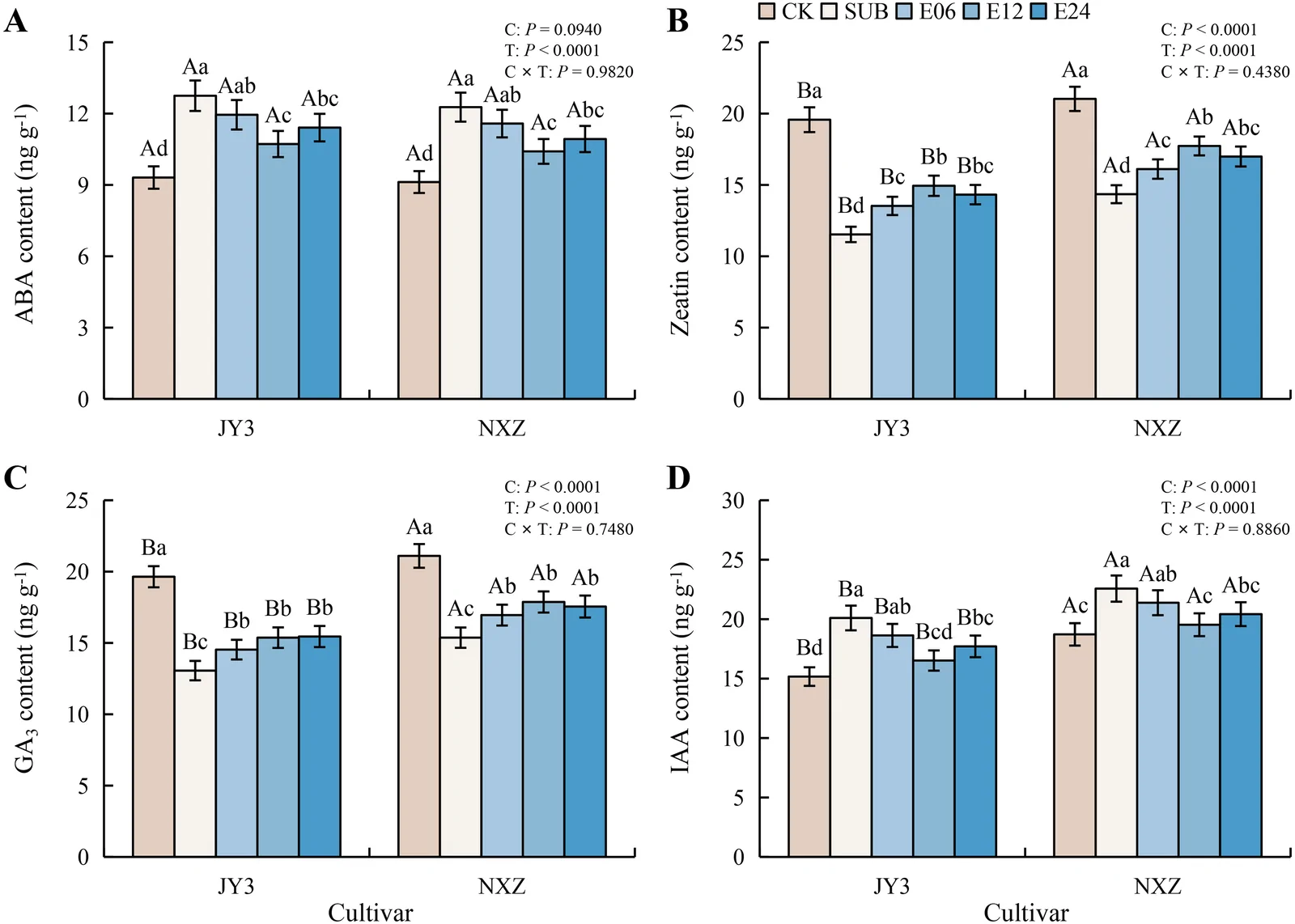
Effects of EBR pretreatment on leaf hormone contents in rice under complete submergence. **(A)** Abscisic acid (ABA) content, **(B)** zeatin content, **(C)** gibberellin A_3_ (GA_3_) content, and **(D)** IAA content. CK, non-submerged control; SUB, complete-submergence control; E06, E12, and E24, complete submergence following pretreatment with 0.06, 0.12, and 0.24 mg L^-1^ EBR, respectively. Lowercase and uppercase letters denote comparisons among treatments within each cultivar and between cultivars within each treatment, respectively. Means sharing a letter within the corresponding comparison do not differ significantly (Duncan’s test, *P* < 0.05). C, T, and C × T denote cultivar, treatment, and their interaction in two-way ANOVA.

### 3.4 Proteomic profiles and functional enrichment in NXZ

TMT-based proteomic analysis of NXZ leaves identified 5,857 proteins, of which 5,186 were quantifiable across the CK, SUB, and E12 treatments (**Supplementary Table S3**). Principal component analysis clearly separated the three treatments, with PC1 and PC2 explaining 53.80% and 23.40% of the total variance, respectively (**Figure 8A**). Biological replicates clustered closely within each treatment, while the heatmap showed distinct treatment-dependent protein expression patterns (**Figure 8B**). Compared with CK, SUB resulted in 1,037 upregulated and 1,046 downregulated differentially expressed proteins (DEPs), whereas the E12 vs SUB comparison identified 1,011 DEPs, comprising 503 upregulated and 508 downregulated proteins (**Figures 8C, D**; **Dataset S1**).

**Figure 8:**
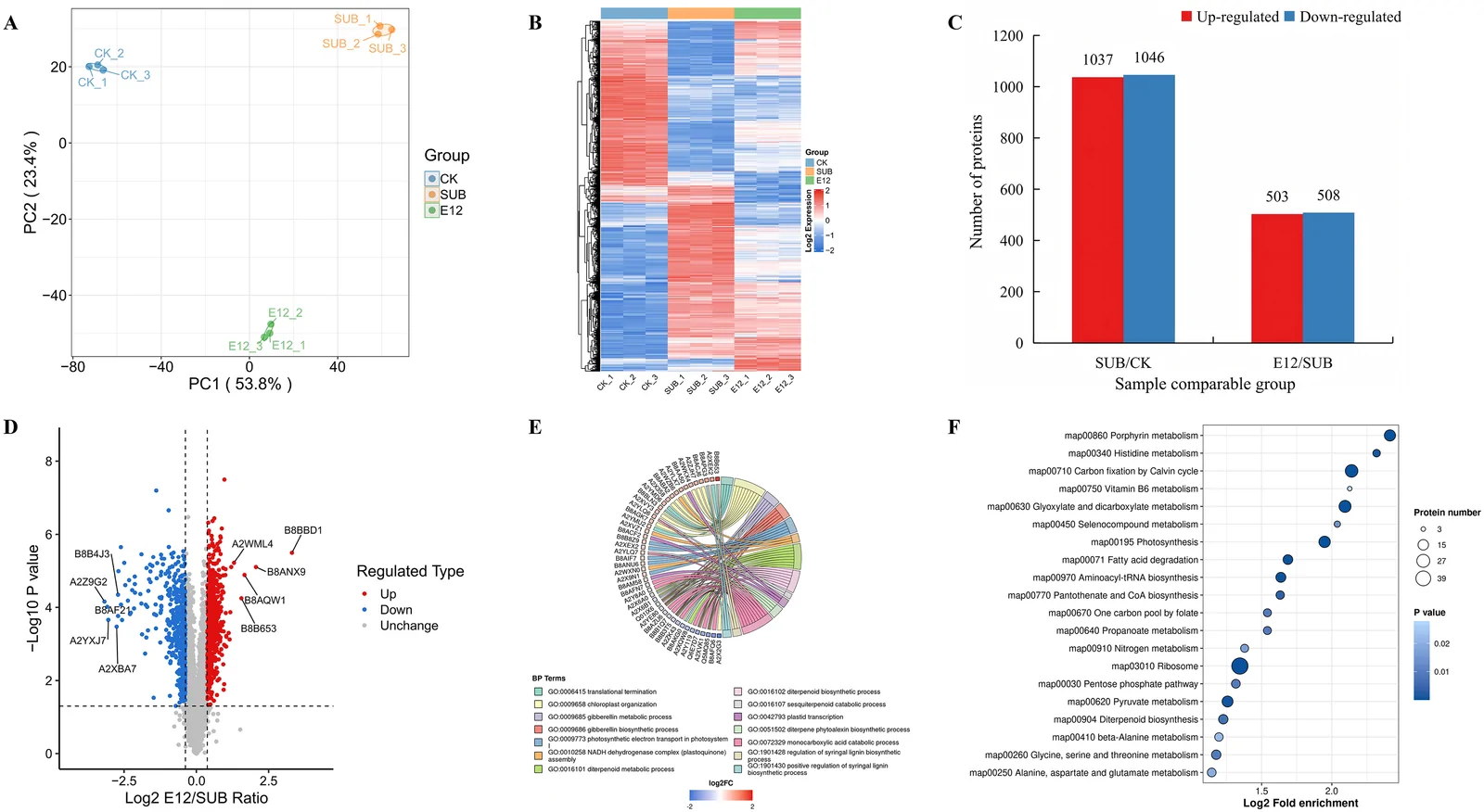
Proteomic responses to EBR pretreatment in the submergence-tolerant rice cultivar under complete submergence. **(A)** PCA of CK, SUB, and E12 samples. **(B)** Hierarchical clustering heatmap of relative protein abundance profiles across the nine samples. **(C)** Numbers of upregulated and downregulated DEPs in the SUB/CK and E12/SUB comparisons. **(D)** Volcano plot of DEPs in the E12/SUB comparison. **(E)** Chord diagram showing enriched Gene Ontology (GO) biological process terms and their associated DEPs in the E12/SUB comparison. **(F)** KEGG pathway enrichment analysis of DEPs in the E12/SUB comparison. CK, non-submerged control; SUB, complete submergence without EBR; E12, complete submergence following foliar pretreatment with 0.12 mg L^-1^ EBR. Each treatment comprised three biological replicates.

Functional annotation of the 503 E12-upregulated DEPs identified 56 proteins associated with energy-related processes (**Supplementary Figure S1A**). These included 10 glycolysis-related proteins, three anaerobic-fermentation proteins, six proteins involved in starch and sucrose metabolism, components of the pentose phosphate pathway, and 32 proteins associated with ATP synthesis. E12 also upregulated 18 stress-responsive proteins, including peroxidases, glutathione S-transferases, and components of the thioredoxin/glutaredoxin system (**Supplementary Figure S1B**). A further 32 upregulated proteins were associated with photosynthesis, including photosystem core components, cytochrome-associated proteins, and Calvin-cycle enzymes (**Supplementary Figure S1C**). GO enrichment analysis of the E12-responsive DEPs identified biological processes related to chloroplast organization, photosynthetic electron transport, monocarboxylic acid catabolism, and gibberellin and diterpenoid biosynthesis (**Figure 8E**). KEGG analysis further identified enriched pathways associated with photosynthesis, carbon fixation by the Calvin cycle, the pentose phosphate pathway, pyruvate metabolism, and diterpenoid biosynthesis (**Figure 8F**). Together, these results showed that E12-responsive proteins were concentrated in energy metabolism, redox-related stress responses, photosynthetic processes, and hormone-associated metabolic pathways.

### 3.5 Phosphoproteomic profiles and functional enrichment in NXZ

Phosphoproteomic analysis of NXZ leaves identified 7,399 phosphorylation sites on 3,354 proteins, of which 3,088 sites on 1,446 proteins were quantifiable across the CK, SUB, and E12 treatments (**Supplementary Table S4**). Principal component analysis clearly separated the three treatments, with PC1 and PC2 explaining 47.80% and 28.20% of the total variance, respectively, while biological replicates clustered within each treatment (**Figure 9A**). Relative to CK, SUB induced 431 upregulated and 480 downregulated differentially phosphorylated sites (DPSs), associated with 318 and 339 phosphoproteins, respectively. The E12/SUB comparison identified 483 DPSs, comprising 358 sites with increased phosphorylation on 280 proteins and 125 sites with decreased phosphorylation on 113 proteins (**Figures 9B, C**; **Dataset S2**).

**Figure 9:**
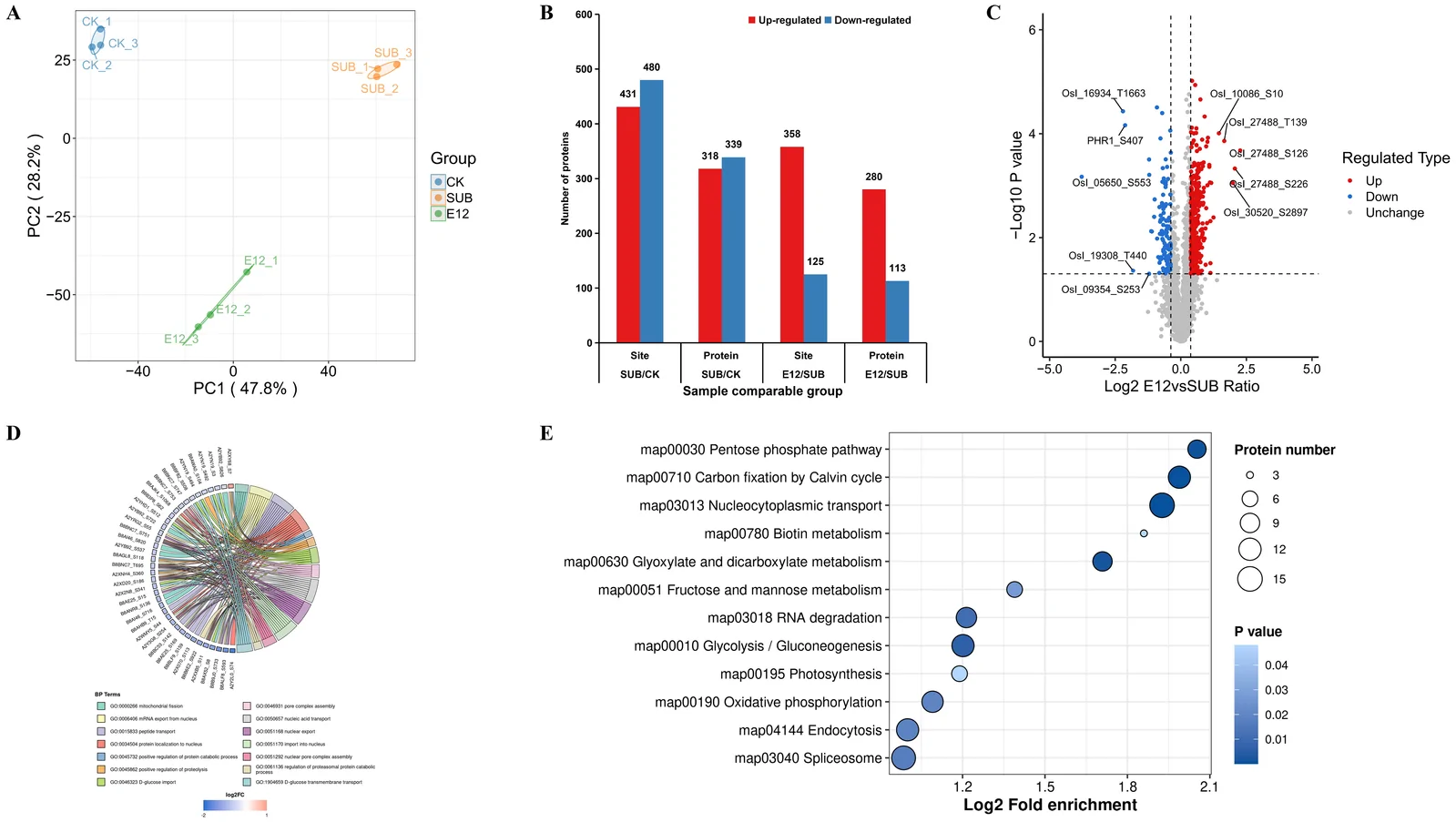
Phosphoproteomic responses to EBR pretreatment in the submergence-tolerant rice cultivar under complete submergence. **(A)** PCA of CK, SUB, and E12 samples. **(B)** Numbers of upregulated and downregulated differentially phosphorylated sites (DPSs) and the corresponding proteins in the SUB/CK and E12/SUB comparisons. **(C)** Volcano plot of DPSs in the E12/SUB comparison. **(D)** Chord diagram showing enriched GO biological process terms and their associated phosphosites in the E12/SUB comparison. **(E)** KEGG pathway enrichment analysis of proteins carrying DPSs in the E12/SUB comparison. CK, non-submerged control; SUB, complete submergence without EBR; E12, complete submergence following foliar pretreatment with 0.12 mg L^-1^ EBR. Each treatment comprised three biological replicates.

Functional annotation of the 280 phosphoproteins containing sites with increased phosphorylation under E12 identified several functional groups related to signaling, metabolism, and cellular homeostasis (**Supplementary Figure S2**). Thirty-three proteins were associated with intracellular signaling, including receptor-like kinases A2XQ61 and B8A706, the MAPK cascade component B8B2Z9, 14-3–3 proteins A2XL95 and A2XUA6, and RING-type E3 ubiquitin ligases A2YXA3 and B8AQB3 (**Supplementary Figure S2A**). Increased phosphorylation was also detected on enzymes involved in sucrose metabolism and glycolysis, including sucrose synthase A2YA91, sucrose-phosphate synthases A2WYE9 and A2YTR9, glyceraldehyde-3-phosphate dehydrogenase A2XU83, and pyruvate kinase B8BM17, together with proteins assigned to the pentose phosphate pathway and fatty acid *β*-oxidation (**Supplementary Figure S2B**). Proteins related to redox regulation, water and ion transport, and membrane trafficking included peroxiredoxin B8AJ36, glutathione reductase A2XAX5, aquaporin A2YKV4, ion transporter A2YPS3, and SNARE protein A2WRE6 (**Supplementary Figure S2C**). E12 also increased phosphorylation at sites on the WRKY protein Q6IEK9, bZIP protein A2Z342, chromatin-associated proteins, molecular chaperones, and proteasome components (**Supplementary Figure S2D**). GO enrichment analysis of proteins associated with E12-responsive DPSs highlighted biological processes involving mRNA export, nuclear transport, nuclear pore complex assembly, protein catabolism, proteolysis, and D-glucose and peptide transport (**Figure 9D**). KEGG enrichment further identified the pentose phosphate pathway, glycolysis/gluconeogenesis, Calvin-cycle carbon fixation, nucleocytoplasmic transport, RNA degradation, photosynthesis, and oxidative phosphorylation (**Figure 9E**). These results showed that the E12-responsive phosphorylation changes were concentrated in intracellular signaling, carbon and energy metabolism, nuclear transport, protein turnover, and cellular homeostasis under complete submergence.

### 3.6 Integrated analysis of protein abundance and site-specific phosphorylation

To examine the relationship between EBR-responsive changes in protein abundance and phosphorylation, the NXZ proteomic and phosphoproteomic datasets were integrated. At the identification level, 1,670 proteins were common to both datasets (**Figure 10A**). For quantitative integration, changes at each phosphosite were compared with changes in the abundance of its corresponding protein, yielding nine regulatory categories (**Figure 10B**). Among the nine regulatory categories, 14 proteins showed concordant increases in protein abundance and phosphorylation, involving 20 sites with significantly increased phosphorylation (**Dataset S3**). This group included fructose-bisphosphate aldolase A2ZBX1, glyceraldehyde-3-phosphate dehydrogenase A2XU83, ferredoxin-NADP reductase A2Y8E0, and peroxidase B8AJE7. Most DPSs, however, occurred without significant changes in protein abundance. In this category, 211 proteins contained 266 sites with increased phosphorylation (**Dataset S4**), including the BRI1-interacting protein B8AFE4 and MAPK-related proteins A2WU94 and B8B2Z9. Conversely, 86 proteins with unchanged abundance contained 96 sites with decreased phosphorylation (**Dataset S5**), including the ABA-signaling regulator SAPK3 (A2ZAB5) and eukaryotic translation initiation factor eIF3a (B8ACZ5). Thus, among the 483 DPSs identified between E12 and SUB, 362 (74.95%) occurred on proteins without significant changes in abundance.

**Figure 10:**
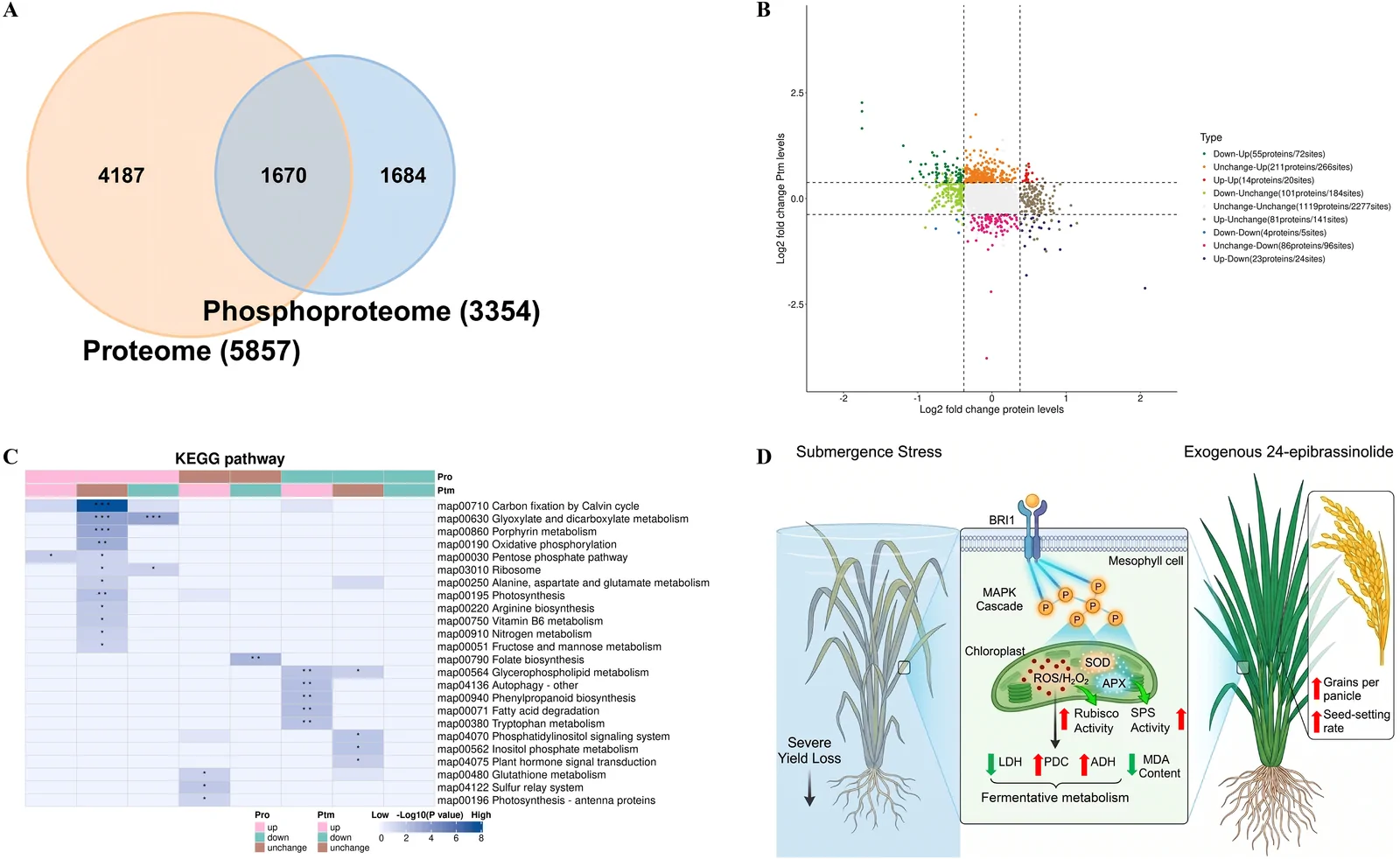
Integrated proteomic and phosphoproteomic responses to EBR pretreatment under complete submergence. **(A)** Overlap of proteins identified in the proteomic and phosphoproteomic datasets. **(B)** Nine-quadrant classification of protein-abundance and phosphorylation changes in the E12/SUB comparison. **(C)** KEGG pathway enrichment of the regulatory categories defined in panel **(B)** Color intensity represents -log_10_(*P* value); one, two, and three asterisks indicate *P* < 0.05, *P* < 0.01, and *P* < 0.001, respectively. **(D)** Proposed working model of EBR-mediated yield protection under complete submergence during rice panicle differentiation. The model integrates physiological responses in both cultivars with proteomic and phosphoproteomic evidence from NXZ. Enhanced antioxidant defense (SOD and APX), reduced ROS and MDA accumulation, higher Rubisco and SPS activities, and fermentative adjustments (LDH, PDC and ADH) are associated with preservation of grain number and seed setting. BRI1/MAPK signaling represents a proposed regulatory framework. Red upward and green downward arrows indicate increases and decreases, respectively, under E12 relative to SUB. ‘P’ denotes protein phosphorylation. Connections among modules indicate proposed functional associations. SPS, sucrose-phosphate synthase. SUB, complete submergence without EBR; E12, complete submergence following pretreatment with 0.12 mg L^-1^ EBR.

Six genes encoding proteins with concordant increases in abundance and phosphorylation were selected for qRT-PCR analysis. These genes were associated with energy metabolism (*OsI_16160* and *OsI_35277*), photosynthesis and defense (*OsI_21320*, *OsI_07630*, and *OsI_05889*), and protein synthesis (*OsI_26912*). RNA-seq and qRT-PCR both showed higher transcript abundance under E12 than under SUB for all six genes, supporting consistent directions of expression change between the two methods (**Supplementary Figure S3**). Category-specific KEGG enrichment identified pathways associated with Calvin-cycle carbon fixation, photosynthesis, oxidative phosphorylation, the pentose phosphate pathway, plant hormone signal transduction, glutathione metabolism, and phosphatidylinositol signaling (**Figure 10C**). A proposed working model integrating the physiological, proteomic, and phosphoproteomic responses is presented in **Figure 10D**. Overall, the integrated analysis distinguished concordant abundance-phosphorylation responses from more prevalent phosphorylation changes occurring without detectable differences in protein abundance.

## 4 Discussion

### 4.1 Enhanced glycolytic and ethanolic fermentation capacity under EBR pretreatment

Complete submergence restricts oxygen-dependent mitochondrial respiration and increases plant reliance on glycolysis and fermentation for ATP production (Leon et al., 2021; Behera et al., 2025). Hypoxia-induced increases in ethanol, acetaldehyde, and lactate have been documented in *Phyllostachys praecox* (Ma et al., 2022). In rice, increased ADH activity accompanied the shift toward anaerobic respiration in tolerant *SUB1* lines under complete submergence (Basu et al., 2021), while broader studies have established fermentative metabolism as an important component of adaptation to oxygen deficiency (Jia et al., 2025; Nishiuchi et al., 2012). Consistent with these reports, SUB reduced SPS activity but increased PFK and PK activities, pyruvate content, and enzymes associated with both lactate and ethanolic fermentation in JY3 and NXZ. E12 partially restored SPS activity while further increasing PFK and PK activities and pyruvate accumulation, suggesting that EBR pretreatment helped sustain carbohydrate metabolism following complete submergence. More importantly, E12 produced contrasting changes in the two fermentation branches. Relative to SUB, E12 significantly reduced LDH activity and lactate content while increasing PDC and ADH activities in both cultivars. The coordinated increases in PDC and ADH activities, together with lower lactate accumulation, are consistent with a greater contribution of ethanolic fermentation, which may support NAD^+^ regeneration for glycolysis and help limit lactate-associated cytosolic acidification. These findings suggest that EBR pretreatment may contribute to the maintenance of carbon metabolism under oxygen limitation through sustained glycolytic capacity and adjustments in fermentation-related enzyme activities.

The physiological evidence for carbon-metabolic adjustment was reinforced by the proteomic and phosphoproteomic profiles of NXZ. Relative to SUB, E12 increased the abundance of multiple proteins associated with glycolysis, the pentose phosphate pathway (PPP), and pyruvate metabolism. These changes were consistent with the higher PFK and PK activities and greater pyruvate accumulation observed at the physiological level. Phosphorylation changes in sucrose-metabolism and glycolytic proteins, together with concordant increases in aldolase and GAPDH abundance and phosphorylation, suggest that carbon-metabolic responses may involve complementary adjustments at both levels. Previous studies have shown that post-translational modifications regulate plant glycolytic enzymes by affecting their catalytic properties, localization, interactions, or stability (O’Leary and Plaxton, 2020). Ethylene accumulation under waterlogging contributes to the regulation of energy metabolism and fermentation-related responses (Chen et al., 2024), while allosteric control and post-translational modifications provide additional regulatory layers in plant central carbon metabolism (Hartman et al., 2023). In the present study, enrichment of PPP- and pyruvate-metabolism components suggests that EBR may coordinate glycolytic carbon processing with cellular redox requirements and anaerobic metabolism. Increased PK phosphorylation coincided with higher PK activity and pyruvate accumulation, highlighting PK as a candidate protein associated with the carbon-metabolic response to EBR. Whether the detected phosphosite directly affects PK function remains to be established. Taken together, the physiological and multi-omics evidence links EBR-mediated maintenance of carbon metabolism under complete submergence in NXZ to multilayered changes in protein abundance and phosphorylation. The accompanying enzyme activity and metabolite patterns were consistent with increased glycolytic capacity and a greater contribution of ethanolic fermentation.

### 4.2 Antioxidant reinforcement and reduced oxidative injury under EBR pretreatment

Oxidative injury was another prominent feature of the submergence response. Concurrent ROS accumulation, lipid peroxidation, and increased relative electrical conductivity indicated that the submergence-induced antioxidant response was insufficient to prevent membrane injury. This pattern agrees with evidence that disturbances in chloroplast and mitochondrial electron transport under oxygen deficiency, together with the oxidative pressure accompanying reoxygenation, promote ROS accumulation, lipid peroxidation, and loss of membrane selectivity (Yuan et al., 2023; Fagerstedt et al., 2024; Renziehausen et al., 2024). The increases in SOD and APX activities and AsA and GSH pools under E12, together with reduced oxidative injury, suggest improved ROS scavenging and maintenance of cellular reducing potential. Comparable protection has been reported in submerged rice following exogenous GSH application (Chi et al., 2023) and in waterlogged soybean treated with EBR (Salah et al., 2022). Recent rice studies have also associated greater submergence tolerance with stronger antioxidant protection and the regulation of glutathione biosynthesis and turnover (Arya and Shylaraj, 2023; Wang et al., 2025a). The smaller relative increases in oxidative injury indicators and stronger antioxidant responses in NXZ were consistent with the contrasting submergence sensitivities of the two cultivars.

The physiological evidence for improved redox homeostasis was paralleled by changes in the NXZ proteome and phosphoproteome. In the E12/SUB comparison, increased abundances of peroxidases, glutathione S-transferases (GSTs), and thioredoxin/glutaredoxin proteins suggest complementary contributions to peroxide removal, detoxification, and cellular redox maintenance. Their increased abundance was therefore consistent with the lower ROS and MDA levels and the higher AsA and GSH contents observed under E12. Integrated transcriptomic and proteomic analyses of waterlogging-tolerant soybean have similarly implicated peroxidase-, dehydroascorbate reductase-, and GST-associated responses in antioxidant protection (Wang et al., 2025b). Recent proteomic analysis of rice exposed to anoxia and subsequent aeration also identified peroxiredoxins, SOD, and other redox-related proteins among the molecular components associated with acclimation to oxygen deprivation and subsequent oxidative stress (Shikov et al., 2025). In addition to these abundance changes, E12 increased the phosphorylation of peroxiredoxin B8AJ36 and glutathione reductase A2XAX5. These phosphorylation changes suggest an additional regulatory layer involving peroxide metabolism and GSH recycling; however, their functional consequences are likely to depend on the modified sites and cannot be equated directly with enzyme activation (Kosova et al., 2021). Taken together, the physiological and multi-omics evidence suggests that E12 alleviated oxidative injury by reinforcing antioxidant capacity and regulating redox-related proteins at both the abundance and phosphorylation levels.

### 4.3 Photosynthetic recovery associated with redox and carbon metabolic adjustments

Photosynthetic inhibition under SUB was associated with concurrent stomatal, biochemical, and redox constraints. The decrease in Gs could restrict CO_2_ diffusion, whereas lower Rubisco activity and SPAD values suggested additional limitations in carboxylation capacity and relative chlorophyll status. The accompanying oxidative and membrane damage may also have contributed to impaired photosynthetic function. Similar coupling among redox imbalance, membrane injury, altered carbon status, and photosynthetic inhibition has been reported in submerged rice (Alpuerto et al., 2016; Basu et al., 2024). Under E12, partial recovery of Pn, Gs, and Rubisco activity in both cultivars coincided with improved antioxidant protection, whereas the increase in SPAD was significant only in NXZ. Improved redox buffering may therefore have supported photosynthetic function as part of a broader metabolic adjustment. E12 also increased the activities of enzymes involved in sucrose metabolism, glycolysis, and ethanolic fermentation, while significantly reducing LDH activity and lactate content in both cultivars. These responses were consistent with increased glycolytic capacity and a greater contribution of ethanolic fermentation. This metabolic adjustment may have helped sustain carbon turnover under oxygen limitation and thereby lessened metabolic constraints on photosynthetic function (Jethva et al., 2022). In NXZ, pathways associated with photosynthesis, Calvin-cycle carbon fixation, glycolysis, the pentose phosphate pathway, and pyruvate metabolism were enriched in the E12 versus SUB comparison, consistent with the observed photosynthetic and carbon-metabolic responses. Increased abundances of photosystem components, cytochrome proteins, and Calvin-cycle enzymes may have supported the observed recovery of photosynthetic function. Integrated proteomic and phosphoproteomic analyses further identified concordant increases in the abundance and phosphorylation of FNR and peroxidase. This coordinated pattern suggests an association among photosynthetic electron transfer, redox regulation, and central carbon metabolism. Because the ferredoxin–FNR system mediates electron transfer downstream of photosystem I and supports carbon assimilation in rice (He et al., 2020), FNR may contribute to the coordination of photosynthetic electron transport and metabolic homeostasis. Its concordant changes in abundance and phosphorylation highlight FNR as a candidate for further functional analysis, although the effect of the identified phosphosite on FNR activity remains to be determined. The partial recovery of photosynthetic performance under E12 was therefore associated with coordinated reinforcement of redox buffering, adjustment of low-oxygen carbon metabolism, and changes in photosynthesis-related protein abundance and phosphorylation.

### 4.4 Physiological responses associated with EBR-mediated yield protection

The reproducible yield response across the two experimental years provides agronomic evidence that EBR pretreatment partially mitigated yield loss caused by complete submergence during panicle differentiation. Under E12, yield recovery was attributable primarily to increases in total grains per panicle and seed-setting rate, whereas effective panicle number, panicle length, and thousand-grain weight did not differ significantly from SUB. Comparable yield-component responses have been reported following excess-water events in rice, with grain number and seed setting among the traits most closely associated with yield loss (Gao et al., 2019). Zhen et al. (2024) similarly reported that short-term flooding during spikelet differentiation reduced grain number without affecting panicle number or individual grain weight. Given the timing of SUB in the present study, the partial recovery of grain number and seed setting under E12 suggests that protection of reproductive development may have contributed to yield preservation. Previous studies have implicated BR homeostasis in the regulation of spikelet differentiation and pollen fertility in young rice panicles, potentially through its effects on antioxidant capacity and nonstructural carbohydrate allocation (Zhang et al., 2019a, b). Several leaf physiological responses were associated with the yield benefit under E12. E12 partially maintained photosynthetic performance while limiting oxidative and membrane injury, responses that may have helped preserve source-leaf function during panicle development. This response is relevant because rice yield formation depends on the coordination of photosynthetic source capacity, reproductive sink demand, and assimilate allocation (Gao et al., 2021). The accompanying adjustments in sucrose metabolism, glycolysis, and fermentation may also have alleviated carbon-metabolic constraints under oxygen limitation. The higher activities of key nitrogen-assimilation enzymes under E12 than under SUB suggest partial preservation of leaf nitrogen-assimilation capacity, which could support reproductive growth through nitrogen and amino-acid allocation to panicles (Guo et al., 2020). The less disrupted leaf hormonal profile under E12 may also be relevant to the hormonal crosstalk regulating rice growth and adaptation to submergence (Ashikari et al., 2025).

In NXZ, the E12/SUB comparison revealed enrichment of pathways associated with photosynthesis, carbon and nitrogen metabolism, and hormone regulation, consistent with the observed physiological responses. Increased phosphorylation of SPS and sucrose synthase suggests that these proteins may be candidates for further studies of leaf sucrose regulation, given the role of sucrose metabolism in source-sink carbon partitioning in rice (Mathan et al., 2021). These findings establish an association between leaf physiological and molecular responses and yield preservation, while the underlying links to developing-panicle processes require further investigation. Previous field research has shown that EBR application at panicle initiation can improve assimilate accumulation and grain yield in rice grown without imposed submergence (Li et al., 2018). Suitable foliar BR concentrations have also been reported to enhance photosynthesis, antioxidant enzyme activities, and grain yield in fragrant rice, although the effective concentration varies among cultivars (Liu et al., 2022). As the experimental design did not include a non-submerged EBR treatment, the EBR × submergence interaction could not be evaluated in this study. Accordingly, the E12 versus SUB comparison supports a benefit of EBR pretreatment under complete submergence, although further investigation is needed to determine whether this benefit is specific to submergence. Further multi-environment field trials are needed to assess the effectiveness and stability of EBR pretreatment against short-duration submergence during panicle differentiation.

## 5 Conclusions

Foliar EBR pretreatment partially mitigated grain yield loss under complete submergence during panicle differentiation in both cultivars. Among the three EBR concentrations evaluated, 0.12 mg L^-1^ resulted in the highest grain yield, primarily through increased grains per panicle and seed-setting rate. E12 strengthened antioxidant defenses, reduced oxidative injury, and partially maintained photosynthetic performance and nitrogen assimilation while moderating hormone imbalance. Changes in enzyme activities and lactate accumulation were consistent with increased glycolytic capacity and a greater contribution of ethanolic fermentation. In NXZ, proteomic and phosphoproteomic differences between E12 and SUB involved photosynthesis, carbon metabolism, and redox regulation, with changes in both protein abundance and phosphorylation. The applicability of these molecular findings to other cultivars and EBR concentrations requires further evaluation. Together, physiological evidence from both cultivars and molecular evidence from NXZ indicate that EBR-mediated yield protection was associated with maintenance of redox balance and photosynthetic carbon assimilation, together with adjustments in low-oxygen carbon metabolism. Nitrogen assimilation and hormonal adjustment may provide complementary support for reproductive development. These findings support further field evaluation of EBR pretreatment as a candidate management option for short-duration submergence during panicle differentiation.

## Data Availability

The datasets presented in this study can be found in online repositories. The names of the repository/repositories and accession number(s) can be found in the article/**Supplementary Material**.

## Supplementary material

Supplementary Data Sheet 1 Information on differentially expressed proteins in the SUB vs CK and E12 vs SUB comparisons in NXZ.

Supplementary Data Sheet 2 Information on differentially phosphorylated sites in the SUB vs CK and E12 vs SUB comparisons in NXZ.

Supplementary Data Sheet 3 Proteins and phosphosites showing concordant increases in protein abundance and phosphorylation under E12 relative to SUB in NXZ.

Supplementary Data Sheet 4 Proteins and phosphosites showing increased phosphorylation without significant changes in protein abundance under E12 relative to SUB in NXZ.

Supplementary Data Sheet 5 Proteins and phosphosites showing decreased phosphorylation without significant changes in protein abundance under E12 relative to SUB in NXZ.
