# Supplementary material for: Integrated physiological, proteomic, and phosphoproteomic analyses of 24-epibrassinolide-mediated yield protection under complete submergence during panicle differentiation in rice
Source: Front Plant Sci. 2026 Sep 30;17:1969548. doi: 10.3389/fpls.2026.1969548 (PMC13662057; doi:10.3389/fpls.2026.1969548)
Supplement: Supplementary file 1 [file DataSheet1.docx]

Supplementary Material

**Table S1.** Physicochemical properties of the experimental soil

| Years | Soil pH | Organic matter  (g kg^-1^) | Total nitrogen  (g kg^-1^) | Available nutrient (mg kg^-1^) | | |
| --- | --- | --- | --- | --- | --- | --- |
|  |  |  |  | N (nitrogen) | P (phosphorus) | K (potassium) |
| 2023 | 7.3 | 18.11 | 0.87 | 87 | 13.7 | 147.9 |
| 2024 | 7.2 | 17.84 | 0.81 | 82 | 13.1 | 141.7 |

**Table S2.** Primers used for qRT-PCR verification of differently expressed genes

| Primer ID | Forward primer | Reverse Primer |
| --- | --- | --- |
| *OsI_16160* | CTCGACCAGAAGTTCGGCAT | GAGCTTCCCCTTGAGGTTCG |
| *OsI_35277* | CTGGCTCCAACAACGAGTCA | TTCACGGCGAGTTCAGATGG |
| *OsI_21320* | ATGGCACATGGTCTTCAGCA | ACGCAGAGTGAAACGGTCTT |
| *OsI_07630* | GCCAATGCTGTCTATGTGCTT | CCTAAGATGATCAGCGGGCA |
| *OsI_05889* | AGAAGTGCAGACACGAAGTTCT | CGGTGAGGTAGGTCCAGAGA |
| *OsI_26912* | AATGACCTGCCTGGCCTTAC | ATGTCCTGCGGTAGGTGTTG |

**Table S3.** Summary of tandem mass tag (TMT)-based quantitative proteomic analysis in the submergence-tolerant rice cultivar NXZ.

| Title | Total spectrums | Matched spectra | Peptides | Unique peptides | Identified proteins | Quantified proteins |
| --- | --- | --- | --- | --- | --- | --- |
| Number | 237,160 | 50,680 | 30,457 | 28,264 | 5,857 | 5,186 |

**Table S4.** Summary of quantitative phosphoproteomic analysis in the submergence-tolerant rice cultivar NXZ.

| Matched spectra | Peptides | Modified peptides | Identified proteins | Quantified proteins | Identified sites | Quantified sites |
| --- | --- | --- | --- | --- | --- | --- |
| 17,225 | 7,503 | 6,734 | 3,354 | 1,446 | 7,399 | 3,088 |

**Figure S1.** Hierarchical clustering of differentially expressed proteins (DEPs) showing increased abundance under E12 relative to SUB in leaves of the submergence-tolerant rice cultivar NXZ. (A) DEPs involved in glycolysis and energy metabolism. (B) DEPs associated with antioxidant defense. (C) DEPs associated with photosynthesis. The color scale indicates standardized relative protein abundance, with blue and red representing lower and higher abundance, respectively. SUB, complete submergence without EBR; E12, complete submergence following pretreatment with 0.12 mg L^-1^ EBR.

**Figure S2.** Hierarchical clustering of differentially phosphorylated sites (DPSs) showing increased phosphorylation under E12 relative to SUB in NXZ leaves. (A) Signal transduction and regulation. (B) Energy metabolism and photosynthesis. (C) Cellular homeostasis and defense. (D) Gene expression and proteostasis. Colors indicate row-standardized phosphorylation levels (Z scores; blue, lower; red, higher). SUB, complete submergence; E12, complete submergence following pretreatment with 0.12 mg L^-1^ EBR.

**Figure S3.** Comparison of RNA-seq and qRT-PCR expression changes for six selected genes in NXZ. Values represent expression fold changes under E12 relative to SUB. qRT-PCR expression was normalized to *OsActin1* and calculated using the 2^-ΔΔ^*^Ct^* method.
